# Effects of SMOF on soil properties, root-zone microbial community structure, metabolites, and maize (*Zea mays* L.) response on a reclaimed barren mountainous land

**DOI:** 10.3389/fmicb.2023.1181245

**Published:** 2023-05-25

**Authors:** Xuqing Li, Daoze Wang, Qiujun Lu, Zhongling Tian, Jianli Yan

**Affiliations:** ^1^Institute of Vegetable, Hangzhou Academy of Agricultural Sciences, Hangzhou, China; ^2^Hangzhou Service Center for Rural Revitalization, Hangzhou, China; ^3^Hangzhou Agricultural and Rural Affairs Guarantee Center, Hangzhou, China; ^4^Key Laboratory of Pollution Exposure and Health Intervention of Zhejiang Province, Interdisciplinary Research Academy (IRA), Zhejiang Shuren University, Hangzhou, China

**Keywords:** SMOF, soil property, microbial community, metabonomics, maize, reclaimed barren mountainous land

## Abstract

**Introduction:**

Maize is the largest crop produced in China. With the growing population and the rapid development of urbanization and industrialization, maize has been recently cultivated in reclaimed barren mountainous lands in Zhejiang Province, China. However, the soil is usually not suitable for cultivation because of its low pH and poor nutrient conditions. To improve soil quality for crop growth, various fertilizers, including inorganic, organic, and microbial fertilizers, were used in the field. Among them, organic fertilizer-based sheep manure greatly improved the soil quality and has been widely adopted in reclaimed barren mountainous lands. But the mechanism of action was not well clear.

**Methods:**

The field experiment (SMOF, COF, CCF and the control) was carried out on a reclaimed barren mountainous land in Dayang Village, Hangzhou City, Zhejiang Province, China. To systematically evaluate the effect of SMOF on reclaimed barren mountainous lands, soil properties, the root-zone microbial community structure, metabolites, and maize response were investigated.

**Results:**

Compared with the control, SMOF could not significantly affect the soil pH but caused 46.10%, 28.28%, 101.94%, 56.35%, 79.07%, and 76.07% increases in the OMC, total N, available P, available K, MBC, and MBN, respectively. Based on 16S amplicon sequencing of soil bacteria, compared with the control, SMOF caused a 11.06–334.85% increase in the RA of *Ohtaekwangia, Sphingomonas, unclassified_Sphingomonadaceae*, and *Saccharibacteria* and a 11.91–38.60% reduction in the RA of *Spartobacteria, Gemmatimonas, Gp4, Flavisolibacter, Subdivision3, Gp6*, and *unclassified_Betaproteobacteria*, respectively. Moreover, based on ITS amplicon sequencing of soil fungi, SMOF also caused a 42.52–330.86% increase in the RA of *Podospora, Clitopilus, Ascobolus, Mortierella*, and *Sordaria* and a 20.98–64.46% reduction in the RA of *Knufia, Fusarium, Verticillium*, and *Gibberella*, respectively, compared with the control. RDA of microbial communities and soil properties revealed that the main variables of bacterial and fungal communities included available K, OMC, available P, MBN, and available K, pH, and MBC, respectively. In addition, LC-MS analysis indicated that 15 significant DEMs belonged to benzenoids, lipids, organoheterocyclic compounds, organic acids, phenylpropanoids, polyketides, and organic nitrogen compounds in SMOF and the control group, among which four DEMs were significantly correlated with two genera of bacteria and 10 DEMs were significantly correlated with five genera of fungi. The results revealed complicated interactions between microbes and DEMs in the soil of the maize root zone. Furthermore, the results of field experiments demonstrated that SMOF could cause a significant increase in maize ears and plant biomass.

**Conclusions:**

Overall, the results of this study showed that the application of SMOF not only significantly modified the physical, chemical, and biological properties of reclaimed barren mountainous land but also promoted maize growth. SMOF can be used as a good amendment for maize production in reclaimed barren mountainous lands.

## 1. Introduction

Maize (*Zea mays* L.) originated in the highlands of Mexico ~8,700 years ago (Piperno et al., 2009), is the second most plentiful crop globally (Ort and Long, 2014); it is also the largest crop produced in China (Gong et al., 2015). However, with the growing population and the rapid development of urbanization and industrialization, achieving grain supply security on limited arable land is a major challenge in contemporary times (Gong et al., 2015). In recent years, the barren mountainous areas were reclaimed for agricultural use in Zhejiang Province, China, to meet the demand for cultivated land (Wang et al., 2017). However, in most situations, reclaimed barren mountainous lands were not suitable for cultivation due to their acidic soil and nutrient-poor conditions (Li et al., 2023). Therefore, to develop the mountainous maize industry on reclaimed mountainous lands in Zhejiang Province, China, it is necessary to find effective measures to improve soil quality for the growth of maize.

In the last decades, to meet the requirements of crops, many farmers have resorted to using inorganic fertilizers (including commercial compound fertilizers, CCF). However, prolonged and excessive use of inorganic fertilizers has resulted in soil acidification, hardening, ecosystem degradation, environmental pollution, and so on (Lam et al., 2012; Hu et al., 2014; Roy et al., 2014; Gregorich et al., 2015; Yan et al., 2021). It is well-known that the quality of soil is affected by various factors (including physical, chemical, and biological properties). In the case of newly reclaimed lands, the quality of the soil can be greatly improved by increasing the use of organic fertilizers (such as crop residues, livestock manure, biogas liquid, and so on), which not only modify the physical and chemical properties of the soil but also have a great influence on the microbial communities (Li et al., 2021b, 2022a,b). For example, Li et al. (2021b) indicated that the soil quality was ameliorated by commercial organic fertilizers (COF), which caused a significant increase in pH, organic matter contents (OMC), available phosphorus (P), total nitrogen (N), alkaline hydrolysis N, total bacterial numbers, microbial biomass carbon (MBC), and a greatly increased height and total dry weight of maize seedlings in newly reclaimed lands compared with the control. Li et al. (2022b) showed that two kinds of COF, including PMMR-OF and SM-OF, not only significantly increased the weight of air-dried corn on newly reclaimed lands but also significantly changed the microbiota and chemical properties of corn soil. Li et al. (2022a) discovered that COF, SM, and MR not only significantly changed the pH of the soil but also increased the OMC considerably (in particular SM) and also caused a differential change in the bacterial and fungal community of the newly reclaimed lands.

Sheep manure organic fertilizer (SMOF) has recently been widely used on reclaimed barren mountainous lands in Hangzhou City, Zhejiang Province, China. Based on our previous research, organic fertilizer-based sheep manure had a beneficial effect on the growth of maize by improving the soil quality of newly reclaimed lands (Li et al., 2022b). However, the specific relationships among soil, microbes, and maize on reclaimed barren mountainous lands after applying SMOF were unclear. Thus, the aim of the study was to systematically evaluate the effect of SMOF on maize growth, root-zone soil properties, microbial community structure, and metabolites in reclaimed barren mountainous lands. In addition, we examined the correlation between soil properties, the microbial community structure, the correlation between differentially expressed metabolites (DEMs), and related microbes. This study provided a scientific basis for applying SMOF to promote maize cultivation on reclaimed barren mountainous lands in the future.

## 2. Materials and methods

### 2.1. Experimental design

The field experiment was carried out on a reclaimed barren mountainous land in Dayang Village (29°25′36″N; 119°28′52″E; 200 m above sea level), Hangzhou City, Zhejiang Province, China. The soil type was acidic red with gravel, based on the soil classification system of the FAO-UNESCO, and the initial properties of the top 20 cm of soil were as follows: pH 5.94, OMC 1.49%, total N 0.98 g/kg, available P 50.64 mg/kg, and available K 144.85 mg/kg.

Three kinds of fertilizers were used in the field experiment: (1) sheep mature organic fertilizer (SMOF, mostly consisting of sheep manure, NPK ≥ 4%, OMC ≥ 30%) was provided by Hangzhou Nanwuzhuang Soil Fertilizer Co., Ltd. (Hangzhou, China); (2) commercial organic fertilizer (COF, mostly consisting of chicken manure and mushroom residues, NPK ≥ 4%, OMC ≥ 30%) was provided by Jiande Kairong Soil Fertilizer Co., Ltd. (Hangzhou, China); and (3) chemical compound fertilizer (CCF, N-P-K, 16-16-16, NPK = 48%, containing nitrate N and sulfate K) was provided by Shenzhen Batian Ecological Engineering Co., Ltd. (Shenzhen, China). The dosage of SMOF, COF, and CCF in this study was set according to the local farmers' habits. In detail, through the application of different fertilizers to the reclaimed barren mountainous land, the field experiment consisted of four different treatments: (1) SMOF at 1.50 kg/m^2^; (2) COF at 1.50 kg/m^2^; (3) CCF at 75 g/m^2^; and (4) the treatment without any fertilizer was applied as the control.

The field experiment was randomly designed and conducted from 6 April to 2 July 2022. The area of each plot was 24 m^2^ (length 7.5 m and width 3.2 m, respectively), and the planting density of maize was 35 × 50 cm. On 6 April, the top 0–20 cm of the soil of the experimental field was mixed with different fertilizers before planting, and then the seedlings of maize (cultivar “qianjiangtian 1”, provided by Hangzhou Academy of Agricultural Sciences, Hangzhou, China) were planted in the above-mentioned field. In detail, the maize seeds were placed into 50-cell seedling trays containing reclaimed barren mountainous land soil on 1 March and then kept at 25°C and 65−75% relative humidity for 5 weeks. Fresh maize ears and plants were harvested and measured after 87 days of planting. Each treatment had three replicates.

### 2.2. Measure of maize parameters and soil properties

To evaluate the impact of SMOF on the biomass of maize in the reclaimed barren mountainous land, the fresh weight of maize ears and plants was measured using a digital scale (TCS-50, Shanghai Hento Industrial Co., Ltd., Shanghai, China) after 87 days of planting. Growth promotion efficacy (GPE%) was calculated using the following formula: GPE% = (treatment – control)/control × 100%.

Moreover, soil pH, OMC, total N, available P, available K, MBC, and microbial biomass nitrogen (MBN) were detected as previously described (Jackson, 1973; Brookes et al., 1985; Vance et al., 1987; Baran et al., 2019). In detail, when maize ears and plants from each plot were collected, ~1.0 kg of fresh root-zone soil (5–20 cm) was sampled simultaneously. After drying at room temperature and passing through a 0.45-mm sieve to remove fine roots and debris, the soil properties were measured. In brief, soil pH was measured at a soil/distilled water suspension ratio of 1:5 (g/mL) with a pH meter (FE28, Mettler-Toledo, Zurich, Switzerland); the content of organic matter was determined by the K_2_Cr_2_O_7_ oxidation external heating method; total N was determined using an automatic Kjeldahl distillation-titration unit; available P was determined by hydrochloric acid–ammonium fluoride extraction molybdenum–antimony anti–colorimetry; available K was extracted with 1 M ammonium acetate and determined by a flame photometer; MBC and MBN were determined using the chloroform fumigation extraction method. All the treatments had three replicates.

### 2.3. Genome sequencing

When maize ears and plants were collected on 2 July 2022, each plot sampled 20 g of maize root-zone soil and stored it at −80°C. DNA was extracted from the soil samples using the E.Z.N.A^TM^ Mag–Bind Soil DNA Kit (OMEGA, Norcross, GA, USA) according to the manufacturer's protocols. The DNA quality was assessed using a Qubit^®^ 3.0 fluorometer (ThermoFisher Scientific, USA).

The V3–V4 region of maize root-zone bacterial 16S rRNA genes and the ITS1 region of fungal ITS genes were amplified using the universal primers 341F (5′-CCT ACG GGN GGC WGC AG−3′) and 805R (5′-GAC TAC HVG GGT ATC TAA TCC−3′) (Wu et al., 2015), and ITS1F (5′-CTT GGT CAT TTA GGA AGT AA−3′) and ITS2 (5′-GCT GCG TTC TTC ATC GAT GC−3′) (Adams et al., 2013), respectively. The PCR assay volume was 30 μl, including 15 μl 2 × Hieff^®^ Robust PCR Master Mix, 1 μl (10 μM) of each universal primer, 1 μl of DNA template, and 12 μl of ddH_2_O. The PCR thermal protocol generally consisted of an initial denaturation at 94°C for 3 min, 25 cycles of denaturation at 94°C for 30 s, annealing at 55°C for 30 s, extension at 72°C for 30 s, and a final extension at 72°C for 5 min. After the PCR amplicons were purified with Hieff NGS™ DNA selection beads (Yeasen, Shanghai, China), they were pooled in equal amounts, and pair-end (2 × 250 bp) sequencing was accomplished using the Illumina MiSeq™/Hiseq™ system (Sangon Biotechnology Co., Ltd., Shanghai, China).

The bioinformatics analysis of the microbe was conducted following the methodology used in our previous studies (Jiang et al., 2022; Li et al., 2023). In other words, to ensure high data quality, the raw data were preprocessed using PRINSEQ to remove low-quality reads (average quality score <20) (Schmieder and Edwards, 2011) and were merged using PEAR (v0.9.8) (Zhang et al., 2014). After the primers were trimmed with Cutadapt (v1.18) (Martin, 2011), clean reads were analyzed using Usearch with a 97% similarity cutoff to generate operational taxonomic units (OTUs) (Edgar, 2013, 2016). After selection of the representative read of each OTU using the QIIME package (v2020.06) (Caporaso et al., 2010), all 16S rDNA and ITS representative reads were annotated by blasting against the RDP database using the RDP classifier (Wang et al., 2007; Quast et al., 2013), and the UNITE database using BLAST (Altschul et al., 1997; Urmas et al., 2013), respectively.

### 2.4. Metabolomics assay

Maize root-zone soil samples were collected on 2 July 2022, and 10 g of soil from each plot was stored at −80°C. After thawing at 4°C, 0.1 g of each soil sample was extracted in a 1 ML extract solution (methanol: H_2_O = 3:1, with the isotopically labeled internal standard mixture), homogenized at 35 Hz for 4 min, and sonicated for 5 min in an ice-water bath (repeat three times). Then, the samples were incubated at −40°C for 1 h, centrifuged at 4°C for 15 min (12,000 rpm), and the supernatant was transferred into a new glass vial for further analysis. Indeed, the liquid chromatography-mass spectrometry (LC-MS) analyses were performed using a UHPLC system (Vanquish, Thermo Fisher Scientific), and the conditions were set as follows: chromatographic column: waters ACQUITY UPLC HSS T3 column (1.8 μm, 2.1 mm × 100 mm); mobile phase A: 5 mM ammonium acetate and 5 mM acetic acid in the water; mobile phase B: acetonitrile; column temperature: 4°C, injection volume: 2 μl; gradient program: 0–0.5 min, 95% B; 0.5–7 min, 95% B; 7–8 min, 65−40% B; 8–9 min, 40% B; 9–9.1 min, 40−95% B; 9.1–12 min, 95% B. The ESI source conditions were set as follows: sheath gas flow rate, 50 Arb; aux gas flow rate, 15 Arb; capillary temperature, 320°C; full MS resolution, 60,000; MS/MS resolution, 15,000 collision energy as 10/30/60 in NCE mode; spray voltage, 3.8 kV (positive) or 3.4 kV (negative), respectively. The repeatability and reliability of the analysis process were evaluated by inserting one quality control (QC) sample, which was prepared by mixing an equal aliquot of the supernatants from all samples. All the treatments had six replicates. The Orbitrap Exploris 120 mass spectrometer (Orbitrap MS, Thermo) was used to acquire MS/MS spectra, and the obtained data in this study were converted to the mzXML format using ProteoWizard and processed with an in-house program, then an in-house MS2 database (SANGON) was applied for metabolite annotation.

### 2.5. Statistical analysis

The SPSS software (v16.0, SPSS Inc., Chicago, IL, USA) was used to perform one-way variance analysis (ANOVA). Origin software (v2023, Hampton, MA, USA) was employed for analyzing OTUs and alpha diversity indices (including Chao1, Shannon, and the Simpson index). The principal component analysis (PCA) based on beta diversity metrics from the Bray–Curtis metrics was utilized to observe the structural variation of root-zone soil microbes across samples used in this study (Ramette, 2007). The differences in root-zone soil microbes among different samples were tested using permutational multivariate ANOVA (PERMANOVA), with 999 permutations used to calculate *p*-values (Dixon, 2003). To further observe the differentially abundant microbes between groups, linear discriminant analysis effect size (LEfSe) was conducted to discover the differential biomarkers and estimate the biomarkers' effect size (Segata et al., 2011). To investigate the impact of different environmental factors (such as pH, OMC, total N, available P, available K, MBC, and MBN) on microbial community structure, redundancy discriminant analysis (RDA) was performed using Origin (v2022, Hampton, MA, USA). To investigate the impact of different fertilizers on microbial co-occurrence patterns, a Sparcc correlation coefficient among maize root-zone soil microbial communities was measured based on the relative abundance (RA) of OTUs at different treatments (SMOF, COF, CCF, and control). The high RA (>1%) and statistically significant correlations (*p* < 0.01, Sparcc's correlation *N* > 0.5 or <-0.5) among OTU levels were built into the network analysis. To observe the topology of the co-occurrence networks, the network graph analysis was performed based on these measurements, including average degree, modularity, nodes, and edges. To investigate the effect of SMOF on the metabolites, orthogonal projections to latent structures-discriminant analysis (OPLS-DA), volcano plots, heat maps, chord plots, and matchstick analyses on four different treatments were conducted with the MetaboAnalyst 4.0 platform. The screening thresholds for DEMs were set as variable importance in the projection (VIP) > 1 and *p* < 0.05. To investigate the association between DEMs and differential microbes in different treatment groups, a Spearman correlation coefficient among the high RA of maize root-zone soil microbes (top 40 bacteria or fungi at the genus level) and significant DEMs (the largest VIP value, *p* < 0.05) was measured by clustering a hot map (Hollander et al., 2008).

## 3. Results

### 3.1. Impacts of SMOF on maize production

To evaluate the impacts of SMOF on maize production in the reclaimed barren mountainous land, the fresh weight of maize ears and plants was measured after 87 days of planting when harvested. The results showed that SMOF significantly promoted the growth of maize under field conditions (Table 1). Compared to the control, SMOF caused a 12.29 and 10.53% increase in the fresh weight of maize ears and plants, respectively, while COF caused a 9.72 and 21.57% increase, and CCF resulted in a 0.83% reduction and 15.74% increase, respectively. Furthermore, the fresh weight of maize ears was found to be 1.13-fold higher in the SMOF treatment, while in the COF treatment, it was 1.11-fold higher compared to the CCF treatment. These results suggest that SMOF had a greater effect on promoting the growth of maize ears compared to the other treatments (including COF, CCF, and the control).

**Table 1:** Impacts of SMOF on maize growth promotion.

**Treatments**	**Weight of maize ears (kg)**	**GPE%**	**Weight of maize plants (kg)**	**GPE%**
SMOF (1.50 kg/m^2^)	38.52 ± 0.99a	12.29a	47.75 ± 0.88b	10.53b
COF (1.50 kg/m^2^)	37.63 ± 2.40a	9.72a	52.52 ± 1.15a	21.57a
CCF (75 g/m^2^)	34.02 ± 0.88b	−0.83b	50.00 ± 0.83b	15.74ab
Control	34.30 ± 1.84b	–	43.20 ± 1.65c	–

GPE, growth promotion efficacy; SMOF, sheep mature organic fertilizer; COF, commercial organic fertilizer; CCF, chemical compound fertilizer; control, without any fertilizer.

Means are averages ± standard deviations (SD). Different lowercase letters within the same columns reveal the significance among different treatments (*p* < 0.05).

### 3.2. Impacts of SMOF on the pH of the soil, chemical properties, and total biomass

The results indicated that there was no significant difference in the pH of the soil between SMOF and the control, but SMOF significantly increased the OMC, total N, available P, available K, MBC, and MBN (Table 2). In other words, compared with the control, SMOF caused a 0.50% increase, while COF and CCF caused a 1.67 and 7.18% reduction in the soil pH; the soil OMC was significantly increased by 46.10%, 26.24%, and 40.43% by SMOF, COF, and CCF, respectively; SMOF, COF, and CCF caused a significant increase by 28.28%, 20.20%, and 26.26% in total N, respectively; the content of available P and available K was significantly increased by SMOF, COF, and CCF, with a 101.94%, 67.49%, 53.51%, and 56.35%, 20.80%, and 31.80% increase, respectively. In addition, compared to the control, the MBC and MBN values were unaffected by CCF but significantly increased by SMOF and COF, with 79.09%, 66.01% (for MBC), and 76.07%, 63.81% (for MBN) increases, respectively.

**Table 2:** Impacts of SMOF on the pH, chemical properties, and total biomass of maize soil.

**Treatments**	**pH**	**OMC (%)**	**Total N (g/kg)**	**Available P (mg/kg)**	**Available K (mg/kg)**	**MBC (mg/kg)**	**MBN (mg/kg)**
SMOF (1.50 kg/m^2^)	6.02 ± 0.16a	2.06 ± 0.08 a	1.27 ± 0.05a	99.94 ± 7.34a	229.68 ± 7.54a	106.31 ± 4.47a	201.53 ± 2.12a
COF (1.50 kg/m^2^)	5.89 ± 0.09a	1.78 ± 0.06ab	1.19 ± 0.04a	82.89 ± 1.62b	177.45 ± 6.79c	98.56 ± 4.28a	187.50 ± 6.44b
CCF (75 g/m^2^)	5.56 ± 0.19b	1.98 ± 0.03a	1.25 ± 0.06a	75.97 ± 3.18b	193.62 ± 8.37b	62.77 ± 6.06b	116.46 ± 5.34c
Control	5.99 ± 0.11a	1.41 ± 0.11b	0.99 ± 0.08b	49.49 ± 4.09c	146.90 ± 4.47d	59.37 ± 1.25b	114.46 ± 3.15c

OMC, organic matter contents; MBC, microbial biomass carbon; MBN, microbial biomass nitrogen; SMOF, sheep mature organic fertilizer; COF, commercial organic fertilizer; CCF, chemical compound fertilizer; Control, without any fertilizer.

Means are averages ± standard deviations (SD). Different lowercase letters within the same columns reveal the significance among different treatments (*p* < 0.05).

### 3.3. Impacts of SMOF on the microbial community of maize

**Figure 1:**
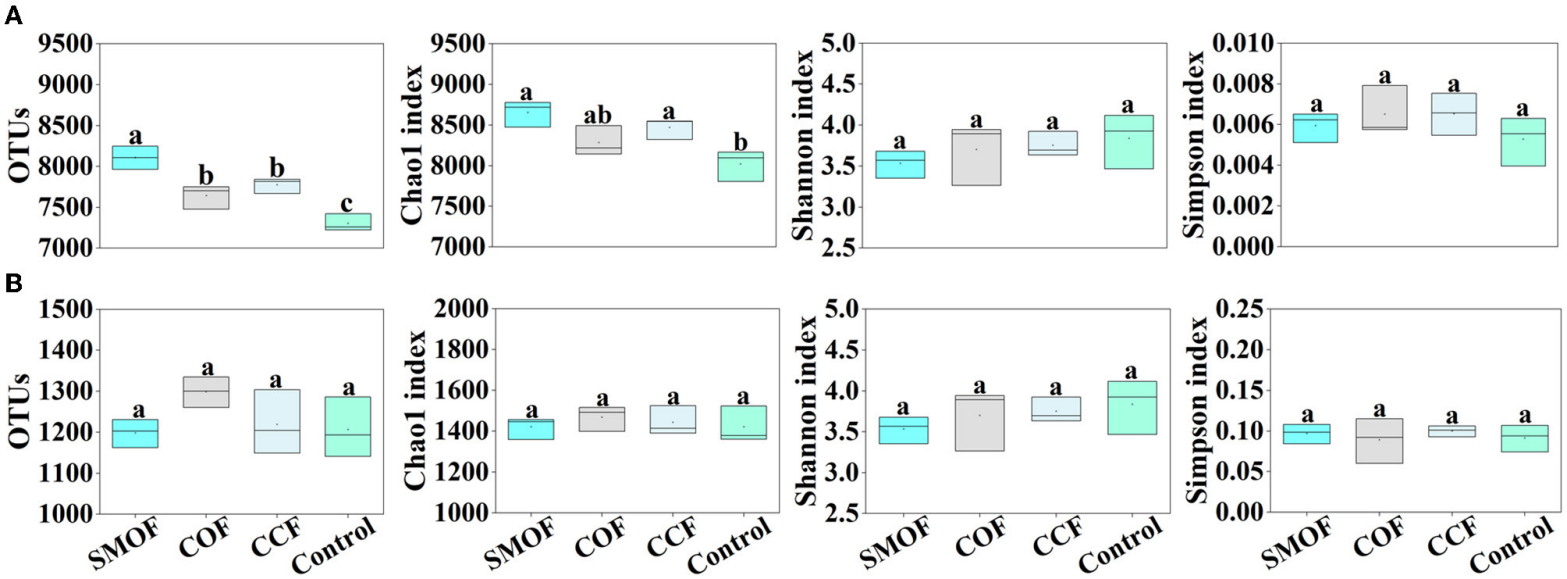
Impacts of SMOF on the OTU distribution, Chao1 richness index, Shannon's diversity index, and Simpson's diversity index of microbes in maize root-zone soil. **(A)** Bacteria, **(B)** fungi. Different lowercase letters above columns indicate statistical differences (*p* < 0.05).

The original microbial sequence data of all soil samples from four different treatments (SMOF, COF, CCF, and the control) were quality-controlled, and a total of 2,109,642 high-quality 16S rRNA gene sequences were obtained from the high-throughput amplicon sequencing. Among them, the high-quality sequences of each sample range from 154,878 to 193,807. A total of 92,480 OTUs from 13 bacterial phyla were identified, and the distribution of OTUs across all four treatments is shown in Figure 1A. Compared with the control, the number of bacterial OTUs was significantly increased by SMOF, COF, and CCF, with a 11.03%, 4.68%, and 6.50% increase, respectively. Similarly, after the raw data were quality-controlled, a total of 4,417,896 high-quality ITS gene sequences were obtained from all soil samples from the four treatments. Among them, the high-quality sequences of each sample range from 215,206 to 475,487. A total of 14,768 OTUs from six fungal phyla were identified, and the distribution of OTUs across all four treatments is shown in Figure 1B. The fungal OTU number was slightly increased (1.70%, 0.67%, and 8.31%, respectively) by SMOF, COF, and CCF compared with the control. Overall, the average bacterial OTU number was 6.65-, 6.33-, 5.99-, and 6.09-fold greater than that of fungi in SMOF, COF, CCF, and the control, respectively. Compared to the control, SMOF caused a greater ratio of bacterial to fungal OTU distribution.

**Figure 2:**
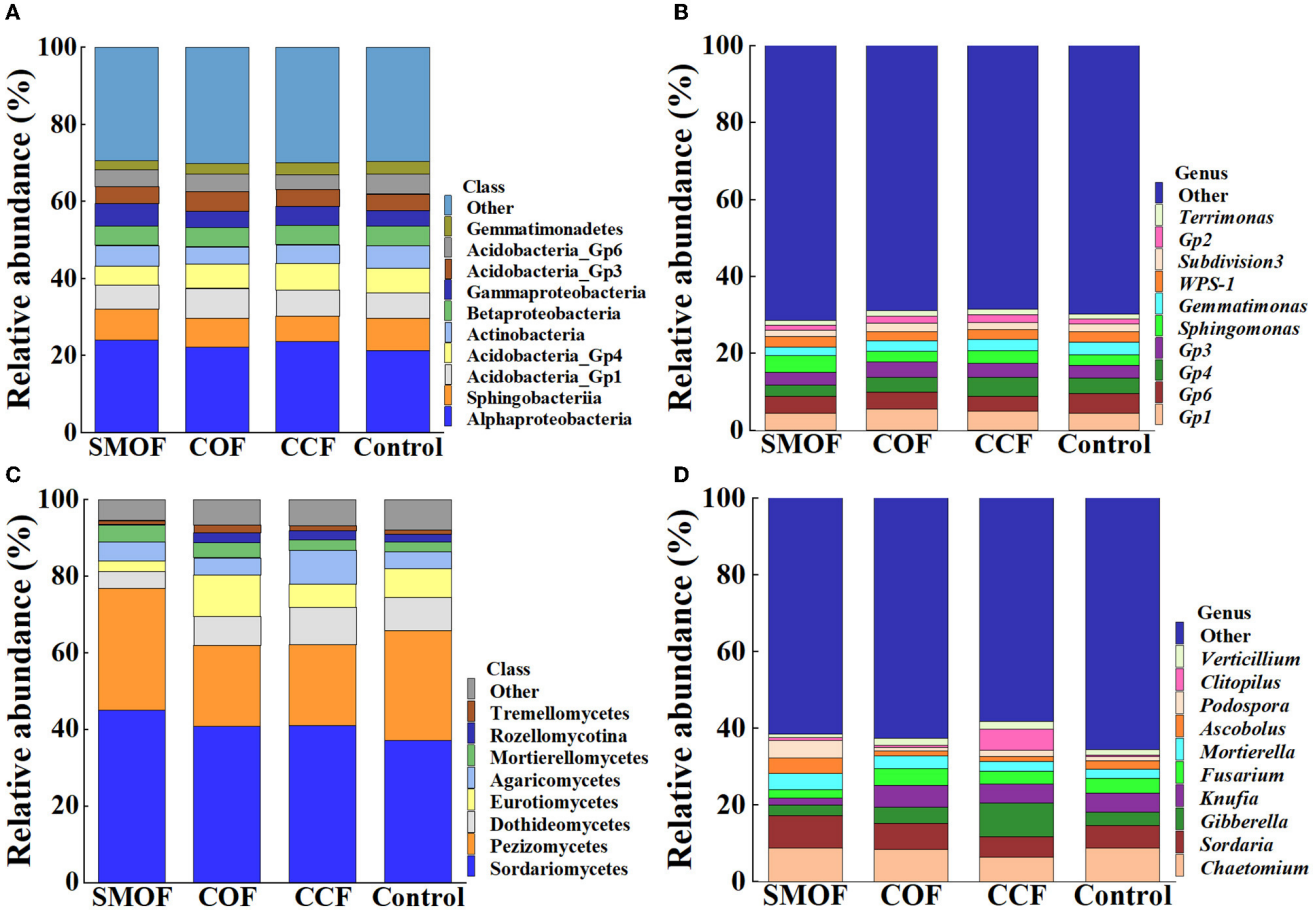
Relative abundance (RA) of the top 10 dominant bacterial **(A, B)** and fungal **(C, D)** classes and genera in four different treatments, respectively.

The RA of the top 10 bacterial classes (Figure 2A) and the whole eight fungal classes (Figure 2C) were observed across all the maize soil samples. Moreover, the top 10 bacterial genera (Figure 2B) and fungal genera (Figure 2D) with an average RA > 1% were highlighted across all the maize soil samples. The results showed that the application of SMOF resulted in significant changes in the microbial community composition of maize root-zone soil in the reclaimed barren mountainous land at the class and genus levels compared to the control. In other words, compared with the control, SMOF significantly increased the RA of *Cytophagia* (153.37%), *Gammaproteobacteria* (39.71%), *Bacilli* (37.97%), *Deltaproteobacteria* (12.47%), *Alphaproteobacteria* (12.20%), and *Saccharibacteria* (11.06%) while also reducing the RA of *Anaerolineae* (66.20%), *Spartobacteria* (35.67%), *Gemmatimonadetes* (30.65%), *Acidobacteria_Gp4* (22.46%), *Subdivision3* (18.35%), and *Acidobacteria_Gp6* (14.27%) at the class level of bacteria. Furthermore, SMOF significantly increased the RA of *Ohtaekwangia* (334.85%), *Sphingomonas* (54.90%), *unclassified_Sphingomonadaceae* (17.76%), and *Saccharibacteria* (11.06%) while reducing the RA of *Spartobacteria* (38.60%), *Gemmatimonas* (30.65%), *Gp4* (27.99%), *Flavisolibacter* (20.17%), *Subdivision3* (18.58%), *Gp6* (14.27%), and *unclassified_Betaproteobacteria* (11.91%) at the genus level of bacteria. Similarly, compared with the control, SMOF significantly increased the RA of *Mortierellomycetes* (70.79%), *Sordariomycetes* (21.02%), *Agaricomycetes* (13.93%), and *Pezizomycetes* (11.19%) while reducing the RA of *Rozellomycotina* (82.16%), *Eurotiomycetes* (63.72%), *Dothideomycetes* (48.33%), and *Tremellomycetes* (15.41%) at the class level of the fungus. Additionally, SMOF significantly increased the RA of *Podospora* (330.86%), *Clitopilus* (86.46%), *Ascobolus* (85.81%), *Mortierella* (74.15%), and *Sordaria* (42.52%) and also reduced the RA of *Knufia* (64.46%), *Fusarium* (42.84%), *Verticillium* (36.22%), and *Gibberella* (20.98%) at the genus level of the fungus. All these results indicated that the changes in the number of specific bacteria and fungi in different treatments might be due to the different nutrients under different fertilizer conditions, and different fertilizers, including SMOF, could affect the abundance of bacteria and fungi in the root-zone soil of maize to regulate the composition of the microbial community.

### 3.4. Impacts of SMOF on microbial α- and β-diversity of maize

The alpha diversity analysis evaluated microbial species richness and diversity within each maize soil sample based on three indices, including Chao1, Shannon, and Simpson (Figure 1). Compared with the control, the bacterial Chao1 index was significantly increased by SMOF (7.91%) and CCF (5.60%), but no significant change was observed in the Shannon and Simpson index of bacterial community among all four different treatments. Moreover, no significant difference was found in the Chao1, Shannon, and Simpson index of fungal community among all four treatments. The results revealed that the bacterial Chao1 index was 5.60-, 5.83-, 5.77-, and 5.65-fold, and the bacterial Shannon index was 1.95-, 1.83-, 1.81-, and 1.78-fold greater than that of fungi in SMOF, COF, CCF, and the control, respectively. The bacterial Simpson index was 0.06-, 0.08-, 0.07-, and 0.05-fold less than fungi in SMOF, COF, CCF, and the control, respectively. Compared to the control, SMOF caused a greater ratio of bacterial and fungal diversity index (Shannon index), but the microbial richness index (Chao1 index) and diversity index (Simpson index) were differentially affected by different treatments. Overall, SMOF could significantly increase the microbial richness of maize root-zone soil in the reclaimed barren mountainous land.

**Figure 3:**
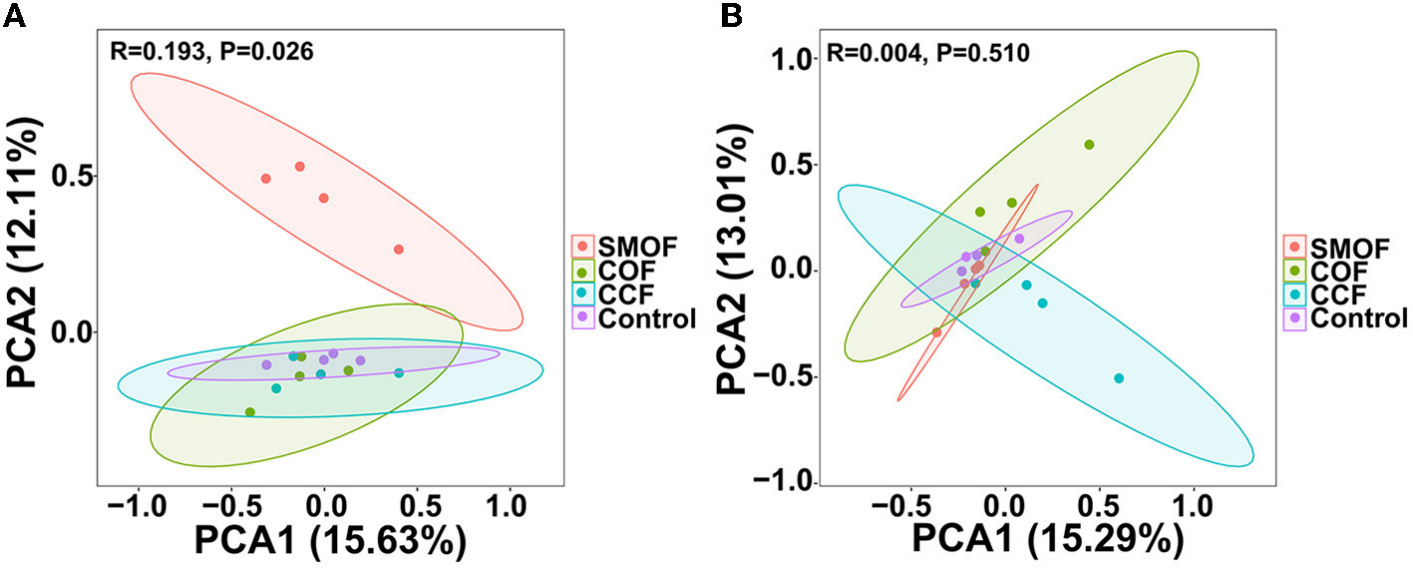
Principal component analysis (PCA) of the maize root-zone bacterial **(A)** and fungal **(B)** communities based on OTU abundance. Ellipses have been drawn for each treatment with a confidence limit of 0.95.

To further study SMOF on maize root-zone microbial communities, principal component analysis (PCA) at the OTU level based on the Bray–Curtis distance was performed (Figure 3). The PCA analysis revealed that the soil root-zone bacterial communities of SMOF, COF, CCF, and the control formed four different groups, and SMOF was well separated from the other three treatments. The first principal component (PCA1) revealed 15.63% of the variability in the bacterial community, and the second principal component (PCA2) explained 12.11%. A PERMANOVA on samples of all four treatments also showed that different fertilizers explained 19.3% of the variation (*p* = 0.026) (Figure 3A). In addition, the PCA analysis of fungal community structure indicated that all samples from four treatments were divided into four groups. However, there was an overlap among all four treatments (Figure 3B). PCA1 and PCA2 explained 15.29 and 13.01% of the total variation in the fungal community, respectively. The bacterial community structure of maize root-zone soil was significantly changed by SMOF, but no significant change was observed in fungal community structure diversity.

### 3.5. Impacts of SMOF on the root-zone microbiome and biomarkers

**Figure 4:**
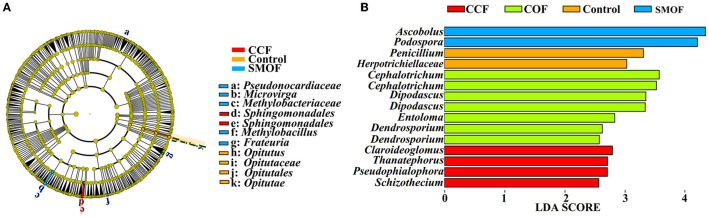
Linear discriminant analysis (LDA) effect size (LEfSe) of the bacterial **(A)** and fungal **(B)** taxa, which identifies the most differentially abundant taxa among SMOF, COF, CCF, and the control in different treatments. Only bacterial taxa with LDA values greater than two and fungal taxa with LDA values >2.5 (*p* < 0.05) are shown.

Linear discriminant analysis (LDA) effect size (LEfSe) was used to identify the biomarkers with significant differences between the maize root-zone soil microbial communities under SMOF, COF, CCF, and the control conditions (Figure 4). Results showed that 11 bacterial biomarkers were found in SMOF, CCF, and the control (Figure 4A). In other words, SMOF was enriched with two types of *Sphingomonadales*. CCF was enriched with *Opitutus, Opitutaceae, Opitutales*, and *Opitutae*. The control was enriched with *Pseudonocardiaceae, Microvirga, Methylobacteriaceae, Methylobacillus*, and *Frateuria*. In addition, a total of 15 fungal biomarkers were obtained in SMOF, COF, CCF, and the control (Figure 4B). Specifically, SMOF was enriched with *Ascobolus* and *Podospora*, COF was enriched with two types of *Cephalotrichum*, two types of *Dipodascus, Entoloma*, and two types of *Dendrosporium*, CCF was enriched with *Claroideoglomus, Thanatephorus, Psedophialophora*, and *Schizothecium*, and the control was enriched with *Penicillium* and *Herpotrichiellaceae*.

**Figure 5:**
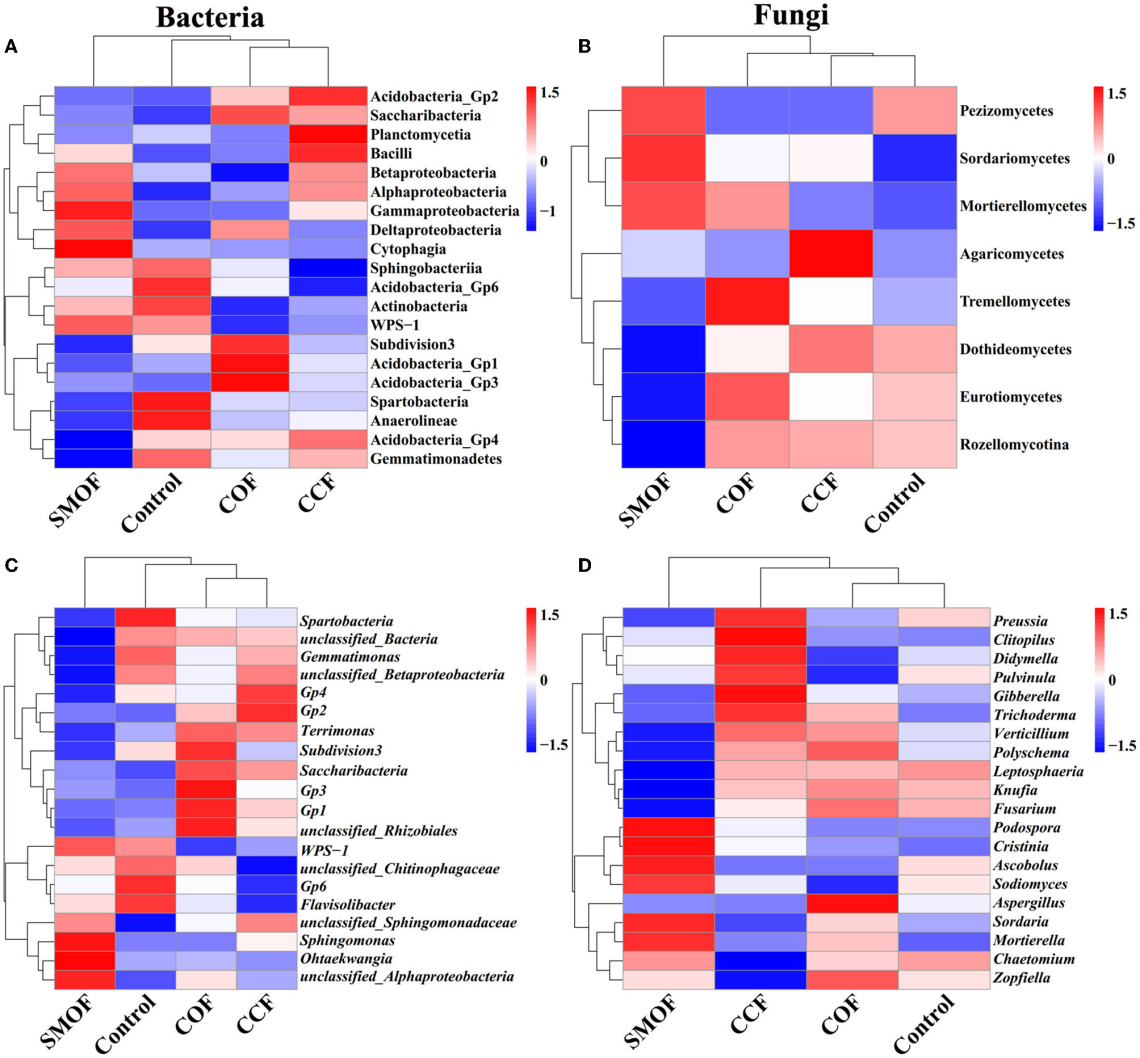
Hierarchical clustering analysis and heat maps of dominant bacterial and fungal community abundance at class **(A, B)** and genus **(C, D)** levels, respectively. The tree plot represents a clustering analysis of the dominant bacteria and fungi at class and genus levels according to their Pearson correlation coefficient matrix and relative abundance, and the upper tree plot represents a clustering analysis of soil samples according to the Euclidean distance of the data, respectively.

Furthermore, the difference in RA composition (class and genus level) of the root-zone microbial community under SMOF, COF, CCF, and the control was visually explained through heat maps (Figure 5). At the class level of bacteria, SMOF was enriched with *Gammaproteobacteria, Cytophagia, Deltaproteobacteria*, and *Alphaproteobacteria* but was reduced with *Spartobacteria, Anaerolineae*, and *Gemmatimonadetes* (*p* < 0.05); COF was enriched with *Acidobacteria_Gp1, Acidobacteria_Gp3, Saccharibacteria*, but was reduced with *Spartobacteria*, and *Anaerolineae* (*p* < 0.05); CCF was enriched with *Acidobacteria_Gp2, Planctomycetia*, and *Bacilli* but was reduced with *Sphingobacteriia* and *Acidobacteria_Gp6* (*p* < 0.05). At the genus level of bacteria, SMOF was enriched with *Sphingomonas, Ohtaekwangia*, and *unclassified_Alphaproteobacteria* but was reduced with *Spartobacteria* and *Gemmatimonas* (*p* < 0.05); COF was enriched with *Gp1, Gp3, unclassified_Rhizobiales*, and *Saccharibacteria* but was reduced with *WPS-1* (*p* < 0.05); CCF was enriched with *Gp2* and *Gp4* but was reduced with *unclassified_Chitinophagaceae, Gp6*, and *Flavisolibacter* (*p* < 0.05). Furthermore, at the class level of the fungus, SMOF was enriched with *Sordariomycetes* and *Mortierellomycetes* but was reduced with *Dothideomycetes, Eurotiomycetes*, and *Rozellomycotina* (*p* < 0.05); COF was enriched with *Tremellomycetes* but was reduced with *Pezizomycetes* (*p* < 0.05); CCF was enriched with *Agaricomycetes* but was reduced with *Pezizomycetes* (*p* < 0.05). At the genus level of the fungus, SMOF was enriched with *Podospora, Cristinia, Sordaria*, and *Mortierella* but was reduced with *Leptosphaeria, Knufia*, and *Fusarium* (*p* < 0.05); COF was enriched with *Aspergillus* but was reduced with *Pulvinula* and *Sodiomyces* (*p* < 0.05); CCF was enriched with *Clitopilus, Gibberella*, and *Trichoderma* but was reduced with *Chaetomium* and *Zopfiella* (*p* < 0.05). The results demonstrated that different fertilizer treatments, such as SMOF, COF, and CCF, had an impact on the presence or absence of specific microbial species, ultimately altering the community structure of the maize root-zone soil.

### 3.6. Impacts of SMOF on the RDA of soil properties and microbial communities

**Figure 6:**
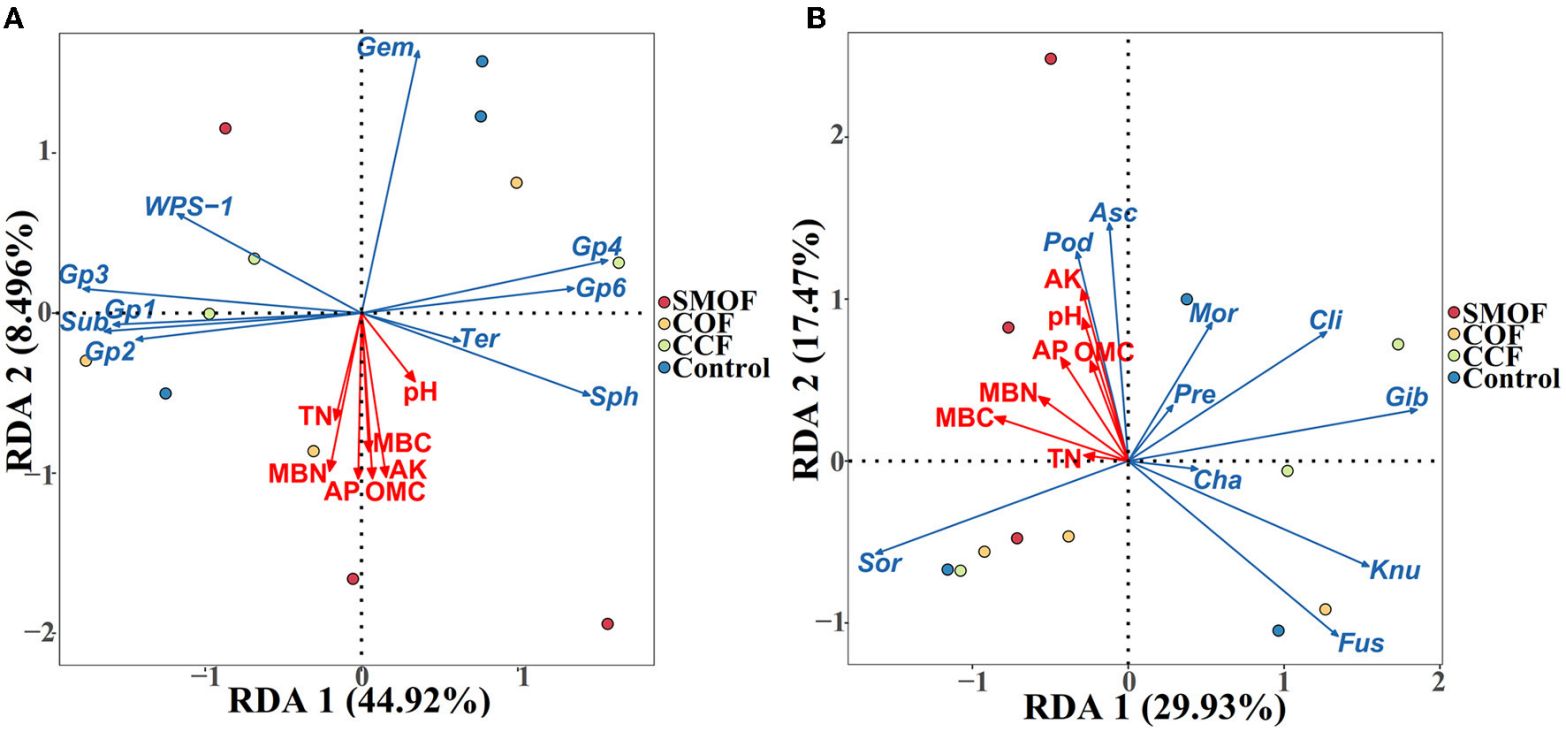
Redundancy discriminant analysis (RDA) of the root-zone microbial community compositions at genus levels with soil properties. **(A)** Bacteria. *Gem, Gemmatimonas*; *Sph, Sphingomonas*; *Sub, Subdivision3*; *Ter, Terrimonas*. **(B)** Fungi. *Asc, Ascobolus*; *Cha, Chaetomium*; *Cli, Clitopilus*; *Fus, Fusarium*; *Gib, Gibberella*; *Knu, Knufia*; *Mor, Mortierella*; *Pod, Podospora*; *Pre, Preussia*; *Sor, Sordaria*. OMC, organic matter contains; TN, total N; AP, available P; AK, available K; MBC, microbial biomass carbon; MBN, microbial biomass nitrogen. Arrows indicate the direction and magnitude of soil properties (pH, OMC, total N, AP, AK, MBC, and MBN) associated with the different bacterial and fungal genera.

Redundancy discriminant analysis (RDA) was used to examine the correlation between environmental factors and microbial communities in maize root-zone soil (Figure 6; Table 3). The results showed that soil properties influenced the composition of microbial communities at the genus level. In addition, 53.42 and 47.40% of the cumulative variance of the root-zone microbial community-factor correction occurred at the bacterial (Figure 6A) and fungal (Figure 6B) genus levels, respectively. The contributions of the four main variables, available K, OMC, available P, and MBN, explained 25.64%, 25.36%, 25.15%, and 23.38% of the bacterial community at the genus level, respectively, while the contributions of the three main variables, available K, pH, and MBC, explained as 32.96%, 23.49%, and 22.67% of the fungal community at the genus level, respectively. All those suggested that available K, OMC, available P, MBN, and available K, pH, MBC were the main factors influencing the bacterial and fungal communities, respectively (Table 3). The results also showed a complex relationship between microbial growth and soil nutrient elements because different soil properties significantly affected microbial community compositions in maize root-zone soil.

**Table 3:** Contribution of the soil environment to bacterial and fungal taxa at the genus level.

**Soil environment**	**Contribution at bacterial genus level (%)**	**Contribution at fungal genus level (%)**
pH	7.29	23.49
OMC	25.36	12.00
Total N	10.87	2.31
Available P	25.15	16.38
Available K	25.64	32.96
MBC	17.76	22.67
MBN	23.38	13.58

### 3.7. Impacts of SMOF on co-occurrence networks of root-zone soil microbes

**Figure 7:**
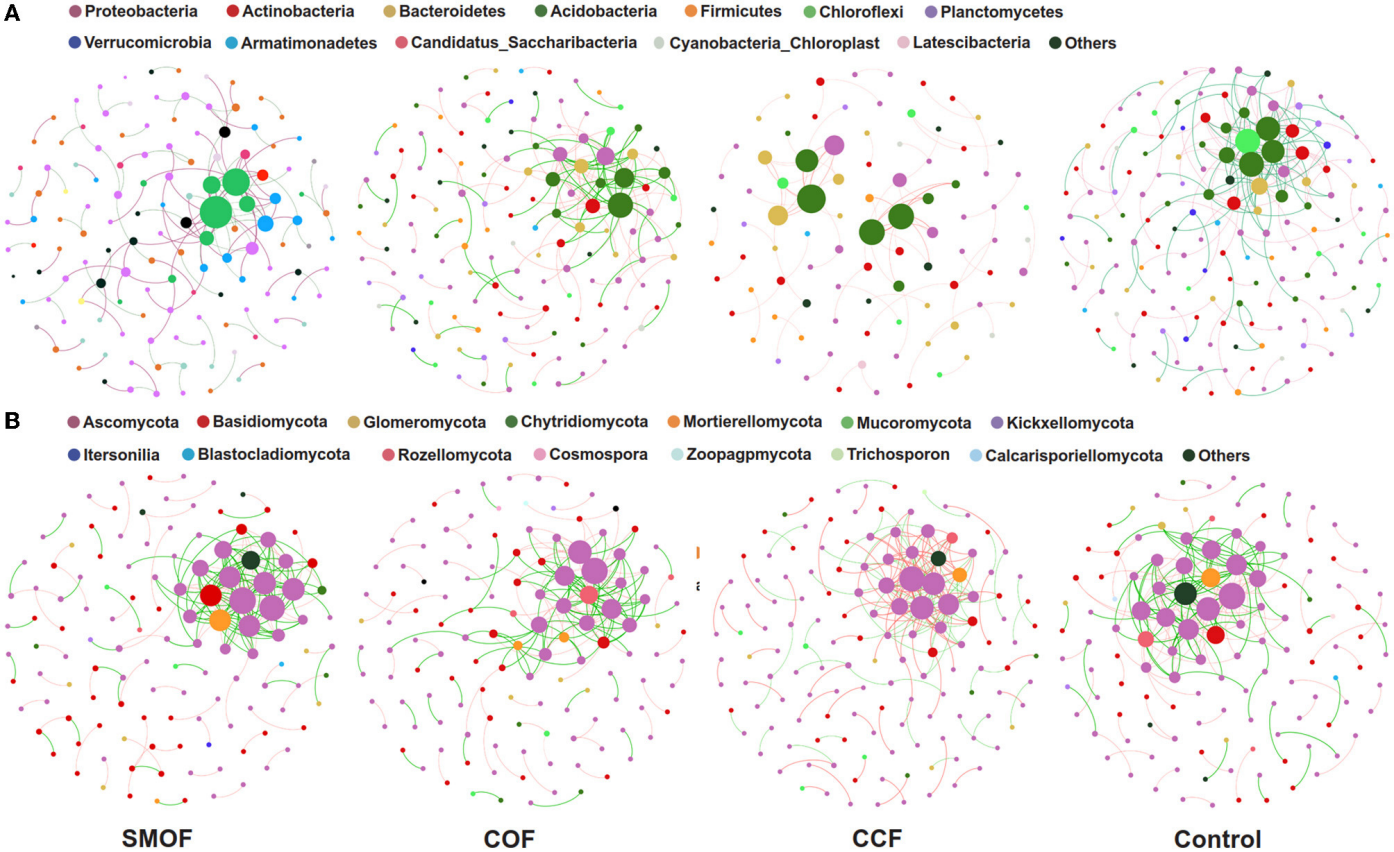
Impacts of SMOF on the co-occurrence patterns of soil bacterial **(A)** and fungal **(B)** communities. Networks were constructed at the OTU level. The size of the nodes (OTUs) represented the relative abundance (RA) of the microbe, and the nodes were colored according to phylum. The red and green lines represented positive and negative correlations, respectively.

Co-occurrence networks were constructed to explore the complexity of connections between maize root-zone soil microbial communities in SMOF, COF, CCF and the control. Moreover, the topological properties of the co-occurrence network were calculated to characterize the differences between different treatments (Figure 7). The bacterial network of SMOF consisted of 175 nodes and 205 edges (97 positive edges, 108 negative edges, and an average degree of 2.343), with modularity of −16.046. The COF, CCF, and control were 175 (positive: 71, negative: 104, average degree 2.500), 79 (positive: 79, negative: 0, average degree 1.756), and 225 (positive: 101, negative: 124, average degree 2.885), consisting of edges and 140, 90, and 156 nodes, respectively, and the modularity was −2.578, 0.798, and −6.410, respectively. Moreover, the fungal network of SMOF consisted of 125 nodes and 207 edges (103 positive edges, 104 negative edges, and an average degree of 3.312), with a modularity of −320.674. The COF, CCF, and control were 189 (positive: 90, negative: 99, average degree 2.953), 205 (positive: 113, negative: 92, average degree 2.929), and 217 (positive: 103, negative: 114, average degree 3.417), consisting of edges and 128, 140, and 127 nodes, respectively, and the modularity was −144.934, 3.317, and 1,311.843, respectively.

### 3.8. Impacts of SMOF on root-zone soil metabolomics

**Figure 8:**
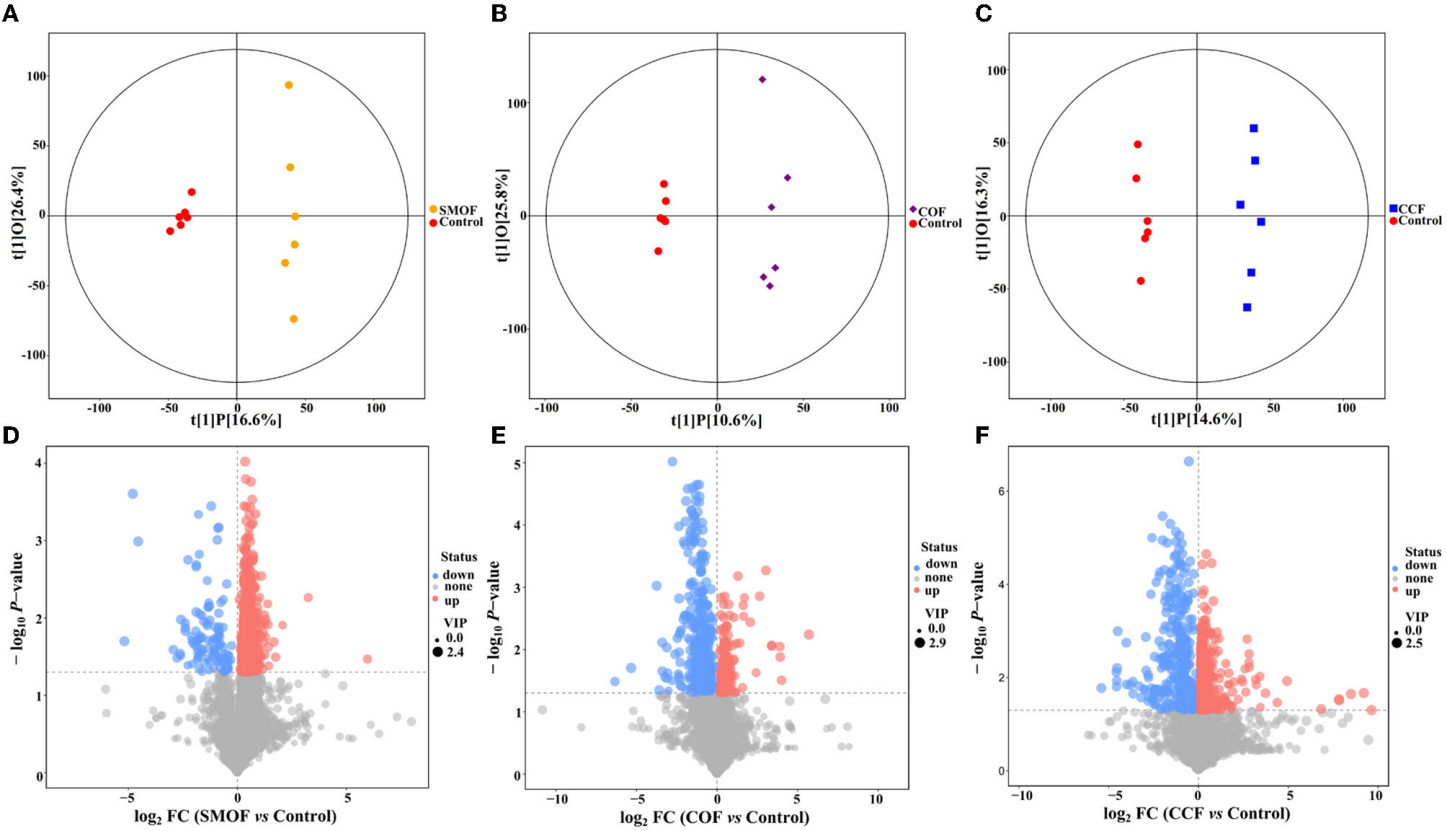
Orthogonal projections to latent structures-discriminant analysis (OPLS-DA) score map of maize root-zone soil of the SMOF **(A)**, COF **(B)**, and CCF **(C)** treatment. Volcano plot of differentially accumulated metabolites in SMOF vs. the control **(D)**, COF vs. the control **(E)**, and CCF vs. the control **(F)**. Each point represents a metabolite with VIP > 1 and *p* < 0.05. A blue point indicates metabolites that are downregulated, a red point indicates metabolites that are upregulated, and a gray point indicates non-significant metabolites.

A total of 10,514 peaks were screened in maize root-zone soil from four different treatments, among which 277 metabolites were identified using LC-MS analysis. Furthermore, a score map of metabolites was constructed using OPLS-DA (Figures 8A–C; Table 4). Results showed that the distribution of different treatments could be effectively separated between SMOF, COF, CCF, and the control. Figure 8A presents the sampling distributions of SMOF treatment and the control in the positive and negative areas of t[1]P, respectively, while the model values of SMOF and the control were *R*^2^*X*(cum) = 0.431, *R*^2^*Y*(cum) = 0.990, and *Q*^2^(cum) = 0.306. Similarly, Figure 8B presents the sampling distributions of COF treatment and the control in the positive and negative areas of t[1]P, respectively, while the model values of COF and the control were *R*^2^*X*(cum) = 0.364, *R*^2^*Y*(cum) = 0.987, and *Q*^2^(cum) = 0.269. Meanwhile, Figure 8C presents the sampling distributions of CCF treatment and the control in the positive and negative areas of t[1]P, respectively, while the model values of CCF and the control were *R*^2^*X*(cum) = 0.310, *R*^2^*Y*(cum) = 0.990, and *Q*^2^(cum) = 0.519. In view of the obvious separation of samples, it could be inferred that the metabolites in control root-zone soil were significantly changed by the application of the SMOF, COF, and CCF. Furthermore, the volcano plot (based on VIP > 1 and *p* < 0.05) also showed that the metabolites in the SMOF, COF, and CCF treatments were significantly different from the control (Figures 8D–F).

**Table 4:** The model parameters of OPLS-DA in different comparative groups.

**OPLS-DA model**	***R*^2^*X* (cum)**	***R*^2^*Y* (cum)**	***Q*^2^ (cum)**
SMOF vs. the control	0.431	0.990	0.306
COF vs. the control	0.364	0.987	0.269
CCF vs. the control	0.310	0.990	0.519

**Figure 9:**
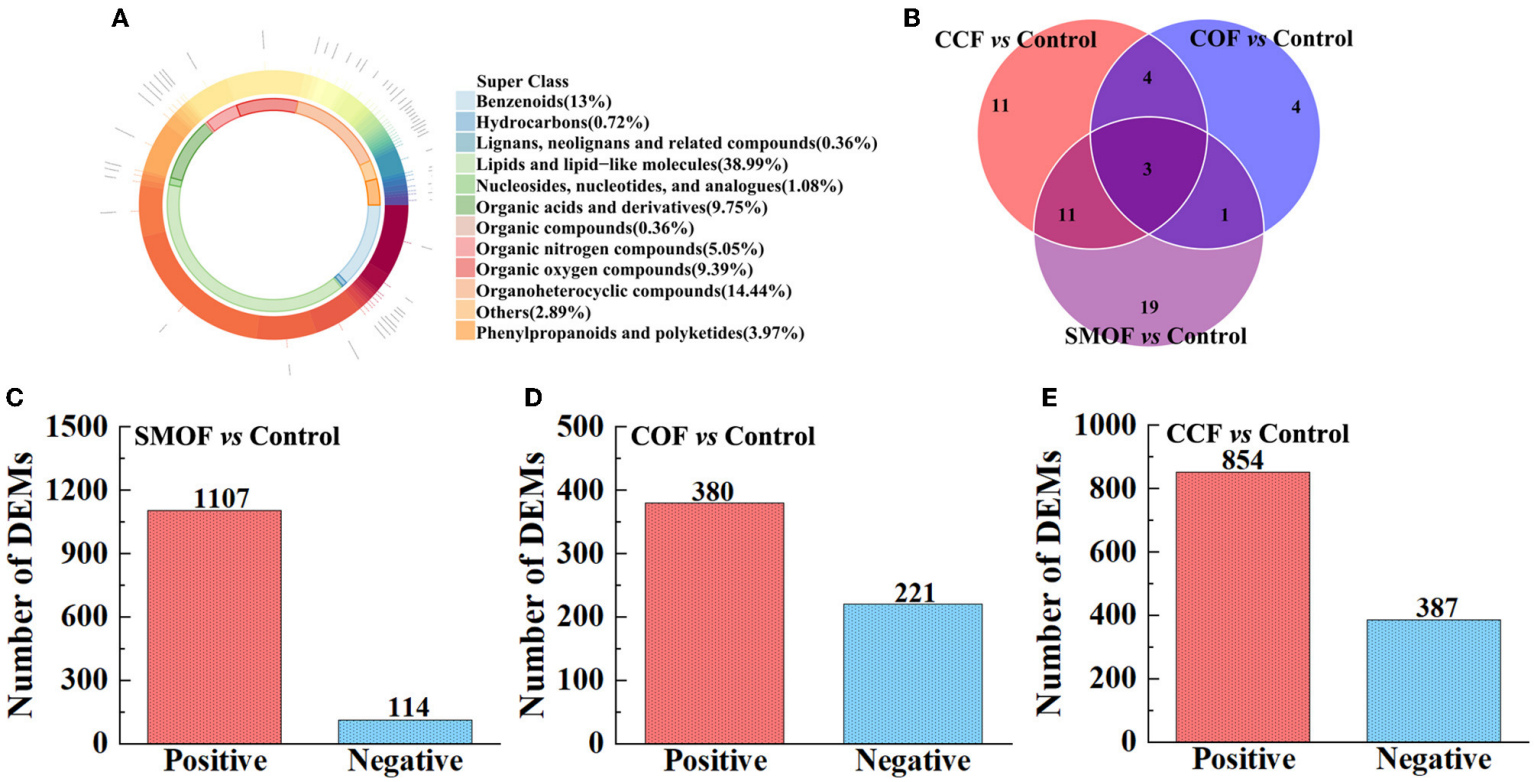
Donut plot of metabolite classification and proportion **(A)**, Venn analysis for three groups **(B)**. The number of differentially expressed metabolites (DEMs) in the groups of SMOF and the control **(C)**, COF and the control **(D)**, and CCF and the control **(E)**.

**Figure 10:**
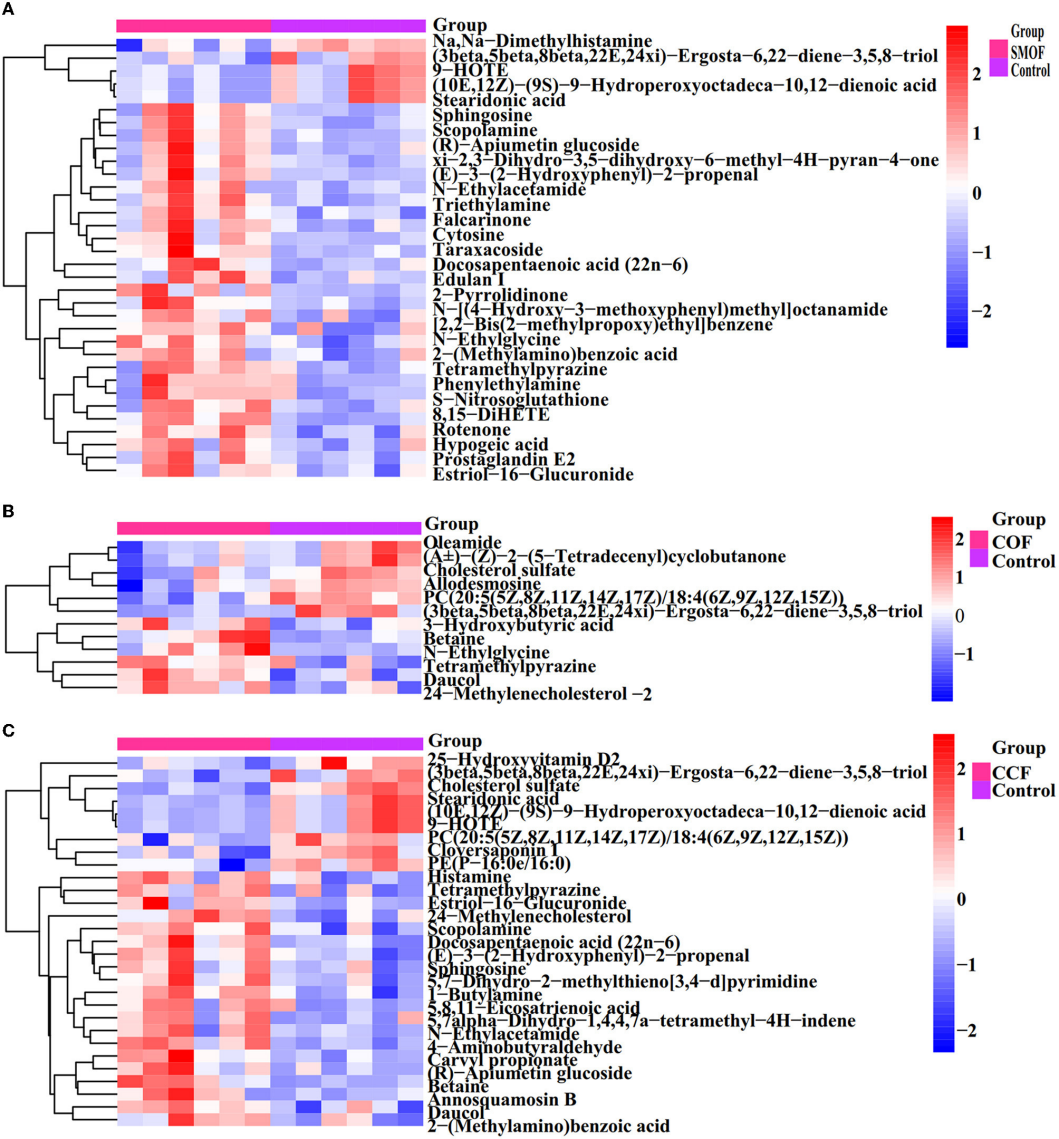
Heat map of hierarchical clustering analysis for groups SMOF, COF, or CCF vs. the control. The tree plot represents a clustering analysis of the differentially expressed metabolites (DEMs) significantly changed by SMOF, COF, or CCF according to their Person correlation coefficient matrix and relative abundance.

All the 277 metabolites mainly refer to lipids and lipid-like molecules (38.99%), organoheterocyclic compounds (14.44%), benzenoids (13%), organic acids and derivatives (9.75%), organic oxygen compounds (9.39%), organic nitrogen compounds (5.05%), and so on (Figure 9A). The results showed differences between the DEMs' numbers of SMOF, COF, or CCF and the control (Figures 9C–E). Indeed, there were 1,221 DEMs in the groups of SMOF and the control, with 1,107 upregulated and 114 downregulated (Figure 9C); 601 DEMs in the group of COF and the control, with 380 upregulated and 221 downregulated (Figure 9D); and 1,241 DEMs in the group of CCF and the control, with 854 upregulated and 387 downregulated (Figure 9E). Furthermore, to observe the change rules for metabolites, the metabolites with significant differences were normalized and shown in the hierarchical clustering heat map (Figure 10). Indeed, compared with the control, 34 DEMs (29 increased by 15.43−132.11%, five decreased by 16.91−73.92%), 12 DEMs (six increased by 14.28−61.68%, six decreased by 25.21−65.13%), and 29 DEMs (20 increased by 5.91−160.39%, nine decreased by 25.51−81.17%) were significantly changed by SMOF, COF, and CCF, respectively (Figures 9B, 10; Table 5).

**Table 5:** A list of differentially expressed metabolites (DEMs) by relative content, VIP, FC, and a *p-*value.

**Compound name**	**Superclass**	**Relative content**			**VIP**	**Log2 (FC)**	***p*-value**	**Regulated**

		**Treatment**		**Control**				
(10E,12Z)-(9S)-9-Hydroperoxyoctadeca-10,12-dienoic acid	Lipids and lipid-like molecules	SMOF	0.133 ± 0.090b	0.465 ± 0.065a	1.584	−1.809	0.019	Down
Rotenone	Phenylpropanoids and polyketides		0.647 ± 0.009a	0.429 ± 0.038b	1.526	0.593	0.037	Up
Taraxacoside	Organic oxygen compounds		0.021 ± 0.003a	0.017 ± 0.002a	1.640	0.287	0.028	Up
Scopolamine	Organic acids and derivatives		0.091 ± 0.014a	0.072 ± 0.006b	1.790	0.338	0.011	Up
Tetramethylpyrazine	Organoheterocyclic compounds		0.369 ± 0.051a	0.254 ± 0.063b	1.576	0.542	0.044	Up
8,15-DiHETE	Lipids and lipid-like molecules		0.752 ± 0.039a	0.506 ± 0.055b	1.488	0.574	0.033	Up
Sphingosine	Organic nitrogen compounds		0.072 ± 0.012a	0.059 ± 0.003a	1.579	0.285	0.042	Up
xi-2,3-Dihydro-3,5-dihydroxy-6-methyl-4H-pyran-4-one	Organoheterocyclic compounds		0.204 ± 0.027a	0.174 ± 0.011a	1.594	0.235	0.028	Up
Stearidonic acid	Lipids and lipid-like molecules		0.041 ± 0.011b	0.159 ± 0.023a	1.409	−1.939	0.021	Down
(E)-3-(2-Hydroxyphenyl)-2-propenal	Phenylpropanoids and polyketides		0.066 ± 0.010a	0.055 ± 0.003b	1.582	0.266	0.044	Up
Morpholine	Organoheterocyclic compounds		0.734 ± 0.086a	0.636 ± 0.036b	1.556	0.208	0.027	Up
Na,Na-Dimethylhistamine	Organic nitrogen compounds		0.172 ± 0.027b	0.207 ± 0.008a	1.644	−0.267	0.023	Down
N-Ethylacetamide	Organic acids and derivatives		0.355 ± 0.058a	0.291 ± 0.007b	1.645	0.285	0.043	Up
Hypogeic acid	Lipids and lipid-like molecules		51.341 ± 2.829a	34.086 ± 1.540b	1.494	0.591	0.033	Up
2-Pyrrolidinone	Organoheterocyclic compounds		0.585 ± 0.079a	0.388 ± 0.072b	1.585	0.594	0.030	Up
Triethylamine	Organic nitrogen compounds		0.204 ± 0.034a	0.165 ± 0.010b	1.545	0.304	0.039	Up
D-Lactic acid	Organic acids and derivatives		0.407 ± 0.060a	0.331 ± 0.050a	1.498	0.297	0.039	Up
Cytosine	Organoheterocyclic compounds		0.240 ± 0.038a	0.184 ± 0.027a	1.628	0.386	0.014	Up
Nortriptyline	Benzenoids		0.021 ± 0.003a	0.017 ± 0.003a	1.519	0.307	0.039	Up
Docosapentaenoic acid (22n-6)	Lipids and lipid-like molecules		1.828 ± 0.118a	1.508 ± 0.039b	1.772	0.278	0.029	Up
Prostaglandin E2	Lipids and lipid-like molecules		0.170 ± 0.027a	0.138 ± 0.014a	1.593	0.295	0.030	Up
Edulan I	Organoheterocyclic compounds		0.021 ± 0.005a	0.015 ± 0.002b	1.827	0.516	0.025	Up
(3beta,5beta,8beta,22E,24xi)-Ergosta-6,22-diene-3,5,8-triol	–		0.010 ± 0.005a	0.020 ± 0.008a	1.347	−0.929	0.036	Down
Phenylethylamine	Benzenoids		0.106 ± 0.013a	0.086 ± 0.012b	1.693	0.306	0.018	Up
Daucol	Organoheterocyclic compounds		0.045 ± 0.010a	0.026 ± 0.007b	1.699	0.789	0.010	Up
S-Nitrosoglutathione	Organic acids and derivatives		0.045 ± 0.010a	0.032 ± 0.010b	1.473	0.503	0.042	Up
(R)-Apiumetin glucoside	Phenylpropanoids and polyketides		0.027 ± 0.004a	0.022 ± 0.001b	1.630	0.298	0.031	Up
N-[(4-Hydroxy-3-methoxyphenyl)methyl]octanamide	–		0.031 ± 0.009a	0.018 ± 0.006b	1.432	0.827	0.034	Up
Falcarinone	Organic oxygen compounds		0.016 ± 0.003a	0.012 ± 0.001b	1.764	0.431	0.011	Up
9-HOTE	Lipids and lipid-like molecules		0.094 ± 0.016b	0.306 ± 0.069a	1.575	−1.697	0.018	Down
Estriol-16-Glucuronide	Lipids and lipid-like molecules		0.022 ± 0.002a	0.017 ± 0.001b	2.135	0.407	0.001	Up
N-Ethylglycine	Organic acids and derivatives		0.059 ± 0.007a	0.051 ± 0.002b	1.610	0.207	0.041	Up
2-(Methylamino)benzoic acid	Benzenoids		0.027 ± 0.003a	0.022 ± 0.004b	1.571	0.303	0.036	Up
[2,2-Bis(2-methylpropoxy)ethyl]benzene	Benzenoids		0.064 ± 0.011a	0.028 ± 0.006b	1.880	1.215	0.020	Up
Betaine	Organic acids and derivatives	COF	4.246 ± 0.617a	3.450 ± 0.226b	2.027	0.279	0.030	Up
Tetramethylpyrazine	Organoheterocyclic compounds		0.372 ± 0.063a	0.254 ± 0.063b	1.919	0.552	0.048	Up
Cholesterol sulfate	Lipids and lipid-like molecules		0.194 ± 0.030b	0.299 ± 0.051a	2.117	−0.626	0.021	Down
3-Hydroxybutyric acid	Organic acids and derivatives		0.011 ± 0.001a	0.009 ± 0.001a	1.874	0.265	0.030	Up
Oleamide	Lipids and lipid-like molecules		6.911 ± 0.365b	9.241 ± 0.804a	1.942	−0.419	0.036	Down
PC(20:5(5Z,8Z,11Z,14Z,17Z)/18:4(6Z,9Z,12Z,15Z))	Lipids and lipid-like molecules		0.013 ± 0.011	0.038 ± 0.008	2.241	−1.520	0.001	Down
(Â ±)-(Z)-2-(5-Tetradecenyl)cyclobutanone	–		0.083 ± 0.008a	0.112 ± 0.011b	1.891	−0.435	0.045	Down
(3beta,5beta,8beta,22E,24xi)-Ergosta-6,22-diene-3,5,8-triol	–		0.008 ± 0.002b	0.020 ± 0.008a	2.334	−1.266	0.015	Down
Daucol	Organoheterocyclic compounds		0.042 ± 0.008a	0.026 ± 0.007b	1.919	0.693	0.016	Up
24-Methylenecholesterol	Lipids and lipid-like molecules		0.012 ± 0.001a	0.011 ± 0.001b	2.125	0.193	0.010	Up
Allodesmosine	Carboxylic acids and derivatives		0.011 ± 0.003b	0.014 ± 0.001a	2.121	−0.436	0.025	Down
N-Ethylglycine	Carboxylic acids and derivatives		0.069 ± 0.016a	0.051 ± 0.002b	1.968	0.429	0.041	Up
1-Butylamine	Organic nitrogen compounds	CCF	0.574 ± 0.023a	0.514 ± 0.028a	2.059	0.158	0.002	Up
25-Hydroxyvitamin D2	Lipids and lipid-like molecules		0.020 ± 0.004b	0.038 ± 0.007a	1.393	−0.965	0.036	Down
(10E,12Z)-(9S)-9-Hydroperoxyoctadeca-10,12-dienoic acid	Lipids and lipid-like molecules		0.091 ± 0.023b	0.465 ± 0.065a	2.069	−2.348	0.018	Down
Betaine	Organic acids and derivatives		4.891 ± 0.790a	3.500 ± 0.226b	2.123	0.483	0.006	Up
Scopolamine	Organic acids and derivatives		0.080 ± 0.004a	0.072 ± 0.006b	1.727	0.153	0.017	Up
Tetramethylpyrazine	Organoheterocyclic compounds		0.394 ± 0.077a	0.254 ± 0.063b	1.631	0.636	0.030	Up
Sphingosine	Organic nitrogen compounds		0.064 ± 0.004a	0.059 ± 0.003b	1.525	0.112	0.047	Up
Cholesterol sulfate	Lipids and lipid-like molecules		0.159 ± 0.035b	0.299 ± 0.051a	2.283	−0.912	0.000	Down
Stearidonic acid	Lipids and lipid-like molecules		0.030 ± 0.008a	0.159 ± 0.023b	2.050	−2.409	0.020	Down
(E)-3-(2-Hydroxyphenyl)-2-propenal	Phenylpropanoids and polyketides		0.061 ± 0.003a	0.055 ± 0.003b	1.944	0.160	0.005	Up
N-Ethylacetamide	Organic acids and derivatives		0.320 ± 0.023a	0.291 ± 0.007b	1.820	0.135	0.029	Up
Cloversaponin I	Lipids and lipid-like molecules		0.025 ± 0.002b	0.051 ± 0.012a	1.577	−1.047	0.016	Down
PC(20:5(5Z,8Z,11Z,14Z,17Z)/18:4(6Z,9Z,12Z,15Z))	Lipids and lipid-like molecules		0.024 ± 0.005b	0.038 ± 0.008a	1.665	−0.675	0.018	Down
Docosapentaenoic acid (22n-6)	Lipids and lipid-like molecules		1.635 ± 0.077a	1.508 ± 0.003b	2.031	0.117	0.005	Up
5,7alpha-Dihydro-1,4,4,7a-tetramethyl-4H-indene	Hydrocarbons		1.087 ± 0.046a	1.027 ± 0.036a	1.724	0.083	0.028	Up
Histamine	Organic nitrogen compounds		0.126 ± 0.007a	0.117 ± 0.006b	1.711	0.115	0.024	Up
4-Aminobutyraldehyde	Organic oxygen compounds		0.566 ± 0.026a	0.512 ± 0.011b	2.206	0.147	0.001	Up
(3beta,5beta,8beta,22E,24xi)-Ergosta-6,22-diene-3,5,8-triol	–		0.009 ± 0.005b	0.020 ± 0.008a	1.210	−1.175	0.016	Down
Carvyl propionate	Lipids and lipid-like molecules		0.380 ± 0.064a	0.170 ± 0.025b	1.951	1.158	0.011	Up
Daucol	Organoheterocyclic compounds		0.040 ± 0.005a	0.026 ± 0.007b	1.566	0.604	0.019	Up
(R)-Apiumetin glucoside	Phenylpropanoids and polyketides		0.025 ± 0.002a	0.022 ± 0.001a	1.750	0.180	0.027	Up
PE(P-16:0e/16:0)	Lipids and lipid-like molecules		0.027 ± 0.007a	0.036 ± 0.004b	1.594	−0.425	0.015	Down
5,8,11-Eicosatrienoic acid	Lipids and lipid-like molecules		1.406 ± 0.392a	0.913 ± 0.108b	1.537	0.622	0.039	Up
24-Methylenecholesterol	Lipids and lipid-like molecules		0.012 ± 0.001a	0.011 ± 0.001b	1.824	0.214	0.009	Up
5,7-Dihydro-2-methylthieno[3,4-d]pyrimidine	Organoheterocyclic compounds		2.054 ± 0.128a	1.872 ± 0.088b	1.799	0.133	0.017	Up
9-HOTE	Lipids and lipid-like molecules		0.067 ± 0.020b	0.306 ± 0.069a	2.048	−2.199	0.018	Down
Estriol-16-Glucuronide	Lipids and lipid-like molecules		0.020 ± 0.003a	0.017 ± 0.001a	1.804	0.257	0.016	Up
Annosquamosin B	Lipids and lipid-like molecules		0.313 ± 0.073a	0.120 ± 0.048b	1.583	1.381	0.036	Up
2-(Methylamino)benzoic acid	Benzenoids		0.027 ± 0.004a	0.022 ± 0.004b	1.594	0.314	0.041	Up

Different lowercase letters within the same rows reveal the significance between different treatments (SMOF, COF, or CCF) and the control (*p* < 0.05).

The results also showed that three DEMs [tetramethylpyrazine, (3beta,5beta,8beta,22E,24xi)-Ergosta-6,22-diene-3,5,8-triol, daucol] were common metabolites in all three treatment groups. One DEM (N-Ethylglycine) was a common metabolite in the groups of SMOF or COF and the control; 11 DEMs [(10E,12Z)-(9S)-9-Hydroperoxyoctadeca-10,12-dienoic acid, scopolamine, sphingosine, stearidonic acid, (E)-3-(2-Hydroxyphenyl)-2-propenal, N-Ethylacetamide, docosapentaenoic acid (22n-6), (R)-Apiumetin glucoside, 9-HOTE, estriol-16-glucuronide, and 2-(Methylamino)benzoic acid] were common metabolites in the groups of SMOF or CCF and the control; four DEMs (betaine, cholesterol sulfate, PC(20:5(5Z,8Z,11Z,14Z,17Z)/18:4(6Z,9Z,12Z,15Z)), 24-Methylenecholesterol) were common metabolites in the group of COF or CCF and the control. Nineteen DEMs (rotenone, taraxacoside, 8,15-DiHETE, xi-2,3-Dihydro-3,5-dihydroxy-6-methyl-4H-pyran-4-one, morpholine, Na,Na-Dimethylhistamine, hypogeic acid, 2-Pyrrolidinone, triethylamine, D-lactic acid, cytosine, nortriptyline, prostaglandin E2, edulan I, phenylethylamine, S-Nitrosoglutathione, N-[(4-Hydroxy-3-methoxyphenyl)methyl]octanamide, falcarinone, and [2,2-Bis(2-methylpropoxy)ethyl]benzene) were unique metabolites in the groups of SMOF and the control; four DEMs (3-Hydroxybutyric acid, oleamide, (Â±)-(Z)-2-(5-Tetradecenyl)cyclobutanone, allodesmosine) were unique metabolites in the group of COF and the control; and 11 DEMs (1-Butylamine, 25-Hydroxyvitamin D2, cloversaponin I, 5,7alpha-Dihydro-1,4,4,7a-tetramethyl-4H-indene, histamine, 4-Aminobutyraldehyde, carvyl propionate, PE(P-16:0e/16:0), 5,8,11-Eicosatrienoic acid, 5,7-Dihydro-2-methylthieno[3,4-d]pyrimidine, and annosquamosin B) were unique metabolites in the group of CCF and the control. All those results indicated that these metabolites might play a crucial role in the response of maize to SMOF, COF, or CCF (Figures 9B, 10; Table 5).

**Figure 11:**
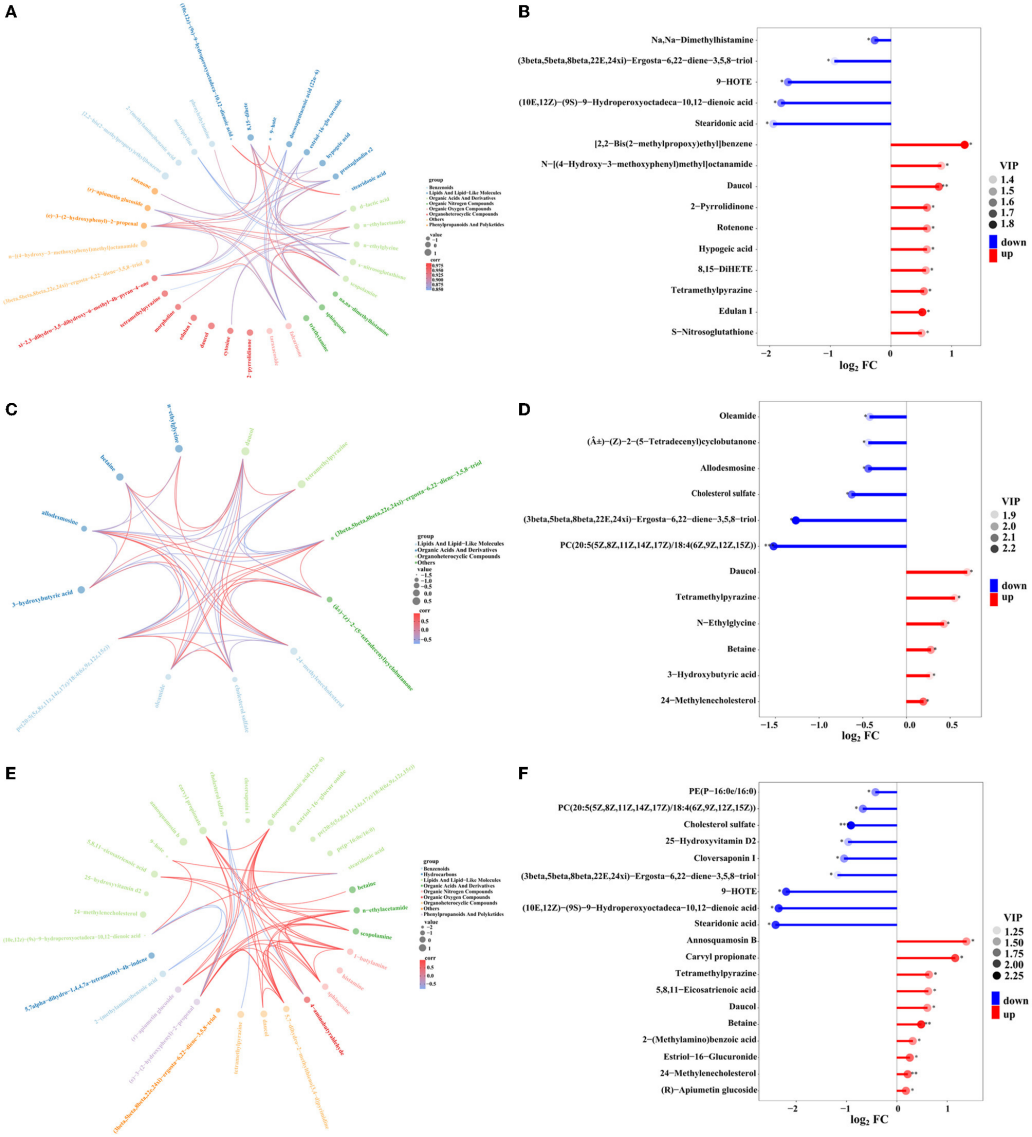
Chord plot analysis for the groups of SMOF and the control **(A)**, COF and the control **(C)**, CCF and the control **(E)**, respectively. Nodes represent variables; text color is associated with different metabolites, and chords represent correlations. Matchstick analysis for the groups of SMOF and the control **(B)**, COF and the control **(D)**, and CCF and the control **(F)**, respectively. The color of the dot represents the size of the VIP value, ^*^represents 0.01 < *p* < 0.05, ^**^represents 0.001 < *p* < 0.01, and ^***^represents *p* < 0.001.

Furthermore, chord plot analysis and matchstick analysis were used to visualize the enrichment analysis of these DEMs in three different treatment groups (SMOF, COF, or CCF, and the control) (Figure 11). The results showed that different metabolites were mostly related to each other. Some were positive correlations, whereas some were negative correlations (Figures 11A, C, E). In detail, there were 10 DEMs significantly upregulated and five DEMs significantly downregulated in the groups of SMOF and the control (Figure 11B); six DEMs significantly upregulated, and the other six DEMs significantly downregulated in the group of COF and the control (Figure 11D); 10 DEMs significantly upregulated and nine DEMs significantly downregulated in the group of CCF and the control (Figure 11F). More attention should be paid to those significant DEMs.

**Figure 12:**
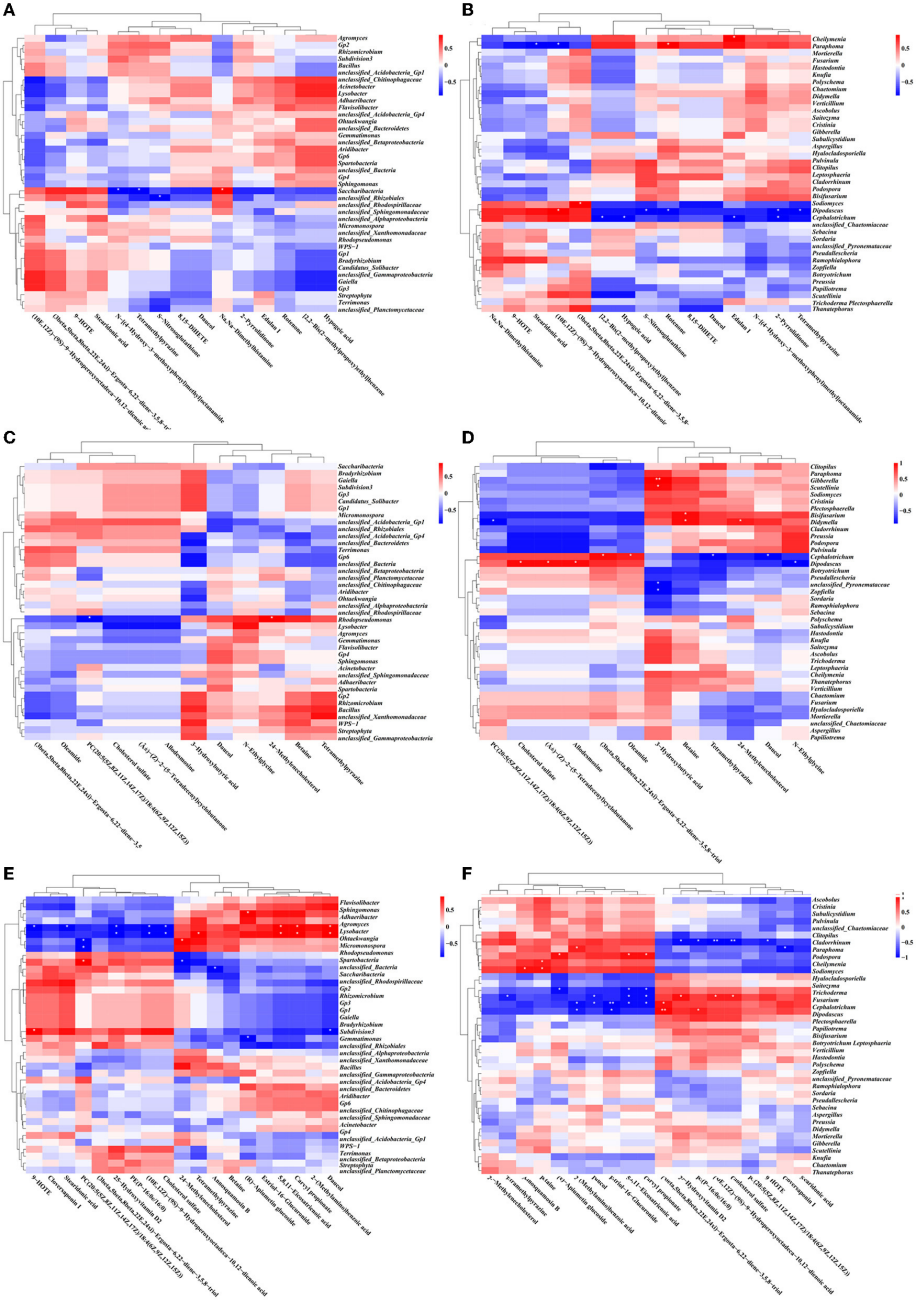
Correlation heat map between DEMs and the related microbe in different treatment groups. Based on the top 40 genera of bacteria: SMOF vs. the control **(A)**, COF vs. the control **(C)**, and CCF vs. the control **(E)**; based on the top 40 genera of fungi: SMOF vs. the control **(B)**, COF vs. the control **(D)**, and CCF vs. the control **(F)**. ^*^Indicated a significant correlation at *p* < 0.05, ^**^indicated a significant correlation at *p* < 0.01.

To further analyze correlative relationships between microbes and metabolites, those metabolites with significant differences were normalized, and the clustering heat map was drawn (Figure 12). The results indicated that in the groups of SMOF and the control, four DEMs (N-[(4-Hydroxy-3-methoxyphenyl)methyl]octanamide, tetramethylpyrazine, Dimethylhistamine, and Nitrosoglutathione) were significantly correlated with two genera of bacteria (*Saccharibacteria, unclassified_Rhizobiales*); meanwhile, 10 DEMs (edulan I, stearidonic acid, (10E,12Z)-(9S)-9-Hydroperoxyoctadeca-10,12-dienoic acid, rotenone, (3beta,5beta,8beta,22E,24xi)-Ergosta-6,22-diene-3,5,8-triol, S-Nitrosoglutathione, 2-Pyrrolidinone, tetramethylpyrazine, [2,2-Bis(2-methylpropoxy)ethyl]benzene, and hypogeic acid) were significantly correlated with five genera of fungi (*Cheilymenia, Paraphoma, Sodiomyces, Dipodascus*, and *Cephalotrichum*). In the group of COF and the control, two DEMs (PC(20:5(5Z,8Z,11Z,14Z,17Z)/18:4(6Z,9Z,12Z,15Z)), and 24-Methylenecholesterol) were significantly correlated with one genus of bacteria (*Rhodopseudomonas*). Meanwhile, 12 DEMs [3-Hydroxybutyric acid, betaine, cholesterol sulfate, (Â±)-(Z)-2-(5-Tetradecenyl)cyclobutanone, Allodesmosine, (3beta,5beta,8beta,22E,24xi)-Ergosta-6,22-diene-3,5,8-triol, oleamide, 24-Methylenecholestero, PC(20:5(5Z,8Z,11Z,14Z,17Z)/18:4(6Z,9Z,12Z,15Z)), tetramethylpyrazine, daucol, and N-Ethylglycine] were significantly correlated with eight genera of fungi (*Gibberella, Scutellinia, Bisifusarium, Didymella, Cephalotrichum, Dipodascus, unclassified_Pyronemataceae*, and *Zopfiella*). In the group of CCF and the control, 13 DEMs [(R)-Apiumetin glucoside, 9-HOTE, stearidonic acid, 25-Hydroxyvitamin D2, (10E,12Z)-(9S)-9-Hydroperoxyoctadeca-10,12-dienoic acid, cholesterol sulfate, 5,8,11-eicosatrienoic acid, carvyl propionate, daucol, tetramethylpyrazine, PC(20:5(5Z,8Z,11Z,14Z,17Z)/18:4(6Z,9Z,12Z,15Z)), 24-Methylenecholesterol, and annosquamosin B] were significantly correlated with nine genera of bacteria (*Adhaeribacter, Lysobacter, Ohtaekwangia, Micromonospora, Rhodopseudomonas, unclassified_Bacteria, Saccharibacteria, Subdivision3*, and *Gemmatimonas*); meanwhile, 16 DEMs [25-Hydroxyvitamin D2, PE(P-16:0e/16:0), (10E,12Z)-(9S)-9-Hydroperoxyoctadeca-10,12-dienoic acid, cholesterol sulfate, 9-HOTE, 2-(Methylamino)benzoic acid, cloversaponin I, (R)-Apiumetin glucoside, 5,8,11-eicosatrienoic acid, carvyl propionate, betaine, annosquamosin B, tetramethylpyrazine, daucol, estriol-16-Glucuronide, and (3beta,5beta,8beta,22E,24xi)-Ergosta-6,22-diene-3,5,8-triol] were significantly correlated with nine genera of fungi (*Cladorrhinum, Paraphoma, Podospora, Cheilymenia, Sodiomyces, Trichoderma, Fusarium, Cephalotrichum*, and *Dipodascus*). Taken overall, the DEMs (organic acids, organoheterocyclic compounds, lipids, and secondary metabolites) produced by the application of different fertilizers (including SMOF) in the root-zone soil of maize may enrich the soil microbe and help coordinate the root-zone microbe.

## 4. Discussion

Cultivated land is the lifeblood and main carrier of food production (Zhou et al., 2021). Cultivated land security is an important guarantee and foundation for food security, social stability, and sustainable development (Zhou et al., 2021). However, with considerable economic development and population growth, various cities have been undergoing rapid urbanization (Zhao, 2016). As the world's most populous country, cultivated lands are occupied on a massive scale in the urban expansion process in China (Tan et al., 2005). Land reclamation is regarded as a viable option to balance occupation and compensation (Li et al., 2023). This practice has been widely adopted worldwide to accommodate urbanization and economic development, especially in developing countries such as China (Hu et al., 2021). However, the soil of reclaimed land usually cannot meet the growth requirements of plants due to poor soil fertility, low soil pH, and high gravel content (Li et al., 2021b).

In most cases, the production capacity of reclaimed land is only 10−30% of the occupied cultivated land in China (Wang et al., 2017), and it often needs 7–10 years of fertilization to reach the level of occupied cultivated land, including acidification adjustment, returning straw to the field, planting green manure, and comprehensive utilization of COF and animal manure (Rendana et al., 2018; Teixeira et al., 2019; Li et al., 2021b). In other words, the content of soil organic matter is highly associated with soil suitability, and the quality of new reclamation land can be effectively improved by increasing the use of organic fertilizers. Indeed, after decades of practice, organic fertilizers have been recognized as valuable soil improvers that could not only improve the physical, chemical, and biological properties of the soil but also increase crop yields (Koocheki and Seyyedi, 2015; Yarami and Sepaskhah, 2015; Qiu et al., 2016; Li et al., 2021b; Yan et al., 2021). In our previous studies, considering the massive consumption in agriculture production, mushroom residue, biogas liquid, vegetable cake, COF, PMMR-OF (organic fertilizer-based pig manure and mushroom residue), and SM-OF (organic fertilizer-based sheep manure) were selected to be applied to new reclamation land, and the results showed that all these organic fertilizers, especially SM-OF, could not only effectively improve the soil quality of new reclamation land by increasing the soil organic matter but also have a greater effect on sweet potato and corn growth compared to the control (Li et al., 2021b, 2022a,b). According to Therios (1996), animal manures differ in nutrient content. It is common knowledge that sheep manure is referred to as cold manure because of its low nitrogen (N) content, and it is also known as a natural slow-release fertilizer with high phosphorus (P), potassium (K), and other essential elements for plant growth. Recently, sheep manure organic fertilizer (SMOF) has been widely used in the agriculture industry on reclaimed barren mountainous land in Dayang Village, Hangzhou City, Zhejiang Province, China. However, the systematically evaluated SMOF on reclaimed barren mountainous land soil properties, root-zone microbial community structure, metabolites, and maize response is very limited.

In this study, SMOF generally significantly promoted maize growth compared to CCF and the control. Moreover, some previous studies also reported that the use of sheep manure could improve the production of plants. For example, El Gammal and Salama (2016) showed that increasing the sheep manure application rate could induce a progressive enhancement of guava trees' growth and fruit set. Amanullah et al. (2019) revealed that the application of N in the form of 50% urea and 50% organic sources (including sheep manure) could significantly increase total rice biomass. Li et al. (2022a) reported that the application of sheep manure resulted in a significantly positive effect on sweet potato production on the new reclamation land.

The soil properties in maize fields were differentially affected by different treatments (SMOF, COF, CCF, and the control), and the effects differed depending on the soil parameters and types of fertilizer. In detail, the soil pH was slightly increased by SMOF but significantly decreased by CCF. A similar observation was observed by Han et al. (2016), which may be due to the H^+^ released into the soil after ${\text{NH}}_{4}^{+}$ used by the plants (Magdoff et al., 1997); the leaching of basic cations such as magnesium (Mg), calcium (Ca), and potassium (K) from the soil (Han et al., 2016); and the proportion of soil microaggregates (<0.25 mm) decreased by the widespread use of CCF (Li et al., 2023). Huang et al. (2009) reported that the soil OMC and total N were widely used as the main parameters for evaluating soil fertility because of their heterogeneous mixtures of organic substances. In our study, the soil OMC and total N were significantly increased by SMOF, COF, and CCF compared with the control. Similar results were obtained by Dong et al. (2012), which may be because the application of organic and chemical fertilizers can promote plant growth and thus return more root residues to the soil (Hyvönen et al., 2008); organic and chemical fertilizers are beneficial to the accumulation of soil organic matter and thus improve the soil's fertility (Dong et al., 2012). The results also showed that available soil P and K contents were significantly affected by different fertilizer treatments (SMOF, COF, and CCF). In particular, SMOF, COF, and CCF led to significantly higher values of soil available P (99.94, 82.89, and 75.97 mg/kg, respectively) and available K (229.68, 177.45, and 193.62 mg/kg, respectively) than the control (49.49, 146.90 mg/kg, respectively). Previous studies have also shown that the application of manure could significantly increase available P (Huang et al., 2010), while available K was the highest in NPK treatments (Dong et al., 2012). In our study, available K was higher in the SMOF treatment than in the other treatments (including CCF), which may be due to the higher concentration of K^+^ in sheep manure (Zhang et al., 2015; Alhrout et al., 2018). Furthermore, MBC and MBN were significantly increased by SMOF and COF compared with the control, but there was no significant difference between CCF and the control. Soil microbial biomass is widely regarded as an important ecological indicator of changes in soil quality and acts as a source of nutrients available for plant uptake and growth (Haripal and Sahoo, 2014; Li et al., 2016). Overall, it can be inferred that SMOF is potentially an effective fertilizer to improve the quality of reclaimed barren mountainous land soil.

Microbial communities inhabiting the maize root zone of three fertilizer treatments and the control were examined using 16S rRNA and ITS gene high-throughput sequencing. We used the number of OTUs, the RA of the top 10 classes, and genera to measure the bacterial and fungal abundance and communities under four different treatment conditions. The results showed that the bacterial OTU number was significantly increased by SMOF, followed by CCF and COF, while the fungal OTU number was slightly increased by CCF, SMOF, and COF, as compared with the control. The results also showed that compared with the control, SMOF significantly increased the RA of *Ohtaekwangia, Sphingomonas, unclassified_Sphingomonadaceae*, and *Saccharibacteria* and reduced the RA of *Spartobacteria, Gemmatimonas, Gp4, Flavisolibacter, Subdivision3, Gp6*, and *unclassified_Betaproteobacteria* at the bacterial genus level; meanwhile, SMOF also significantly increased the RA of *Podospora, Clitopilus, Ascobolus, Mortierella*, and *Sordaria*, and reduced the RA of *Knufia, Fusarium, Verticillium*, and *Gibberella* at the fungal genus level. The change in the number of specific microbes in four different treatments may be due to the different nutrients in different fertilizers. Indeed, more attention should be paid to *Ohtaekwangia, Sphingomonas, Sphingomonadacea, Saccharibacteria, Podospora*, and *Mortierella*. Previous studies have reported that *Ohtaekwangia* showed a significant negative correlation with *Ralstonia solanacearum* (Deng et al., 2021); *Sphingomonas* possessed multifaceted functions, including remediation of environmental contaminations, producing highly beneficial phytohormones, degradation of organometallic compounds, and improving plant growth during stress conditions (Asaf et al., 2020); *Sphingomonadacea* could decompose organic pollutants (Timoshenko et al., 2021); *Saccharibacteria* was pointed out to be involved in hydrocarbon degradation (Figuroa-Gonzalez et al., 2020); *Podospora* produced a wide variety of carbohydrate-active enzymes, including xylanases, cellulases, and lytic polysaccharide monooxygenases (Silar, 2013; Fanuel et al., 2017); *Mortierella* was reported as a plant growth-promoting fungus (PGPF) by mobilizing P from insoluble forms and producing siderophores and phytoregulators (Srinivasan et al., 2012; Wani et al., 2017; Ceci et al., 2018; Ozimek and Hanaka, 2020). Furthermore, we used alpha diversity (Chao1, Shannon, and the Simpson index) and beta diversity (PCA) to measure the microbial species richness and diversity within each maize root-zone soil sample under four different fertilizer treatment conditions. The results showed that SMOF significantly increased the bacterial Chao1 index, but no significant difference was observed in the Chao1 index of fungi and the Shannon and Simpson index of bacteria and fungi. Meanwhile, the PCA analysis revealed that the bacterial community structure of the maize root-zone soil was significantly changed by SMOF (PERMANOVA, *p* < 0.05), and the different fertilizers explained 19.3% (*p* = 0.026) of the variation, but no significant change was observed in fungal community structure diversity. In other words, the richness of the microbe was increased by SMOF, but the diversity was differentially affected by SMOF. The current results align with those of Li et al. (2023), who reported that different fertilizers altered the composition of the pakchoi rhizosphere soil microbial community. Similar results were also obtained by Li et al. (2022c), who reported that three kinds of microbial fertilizer (CMA, TMF, and SMF) could increase the OTU number and bacterial Chao1 index of corn rhizosphere soil in newly reclaimed land, but no significant difference was observed in the Shannon index. Taken overall, the application of SMOF could favor the diversity of beneficial microbes and reshape the root zone microbial abundance distribution by enriching specific soil microbes in maize in a reclaimed barren mountainous land.

To further elucidate the impact of SMOF on microbial groups, LEfSe (LDA > 2 for bacteria or LDA > 2.5 for fungi, *p* < 0.05) was carried out to explore the role of specific microbes in the fertility improvement of reclaimed barren mountainous land soil. A total of 11 bacterial biomarkers were obtained in SMOF, CCF, and the control. Furthermore, the heat map indicated that SMOF could significantly enrich *Sphingomonas, Ohtaekwangia*, and *unclassified_Alphaproteobacteria*. Moreover, a total of 15 fungal biomarkers were collected from four different treatments. The heat map revealed that SMOF could significantly enrich *Podospora, Cristinia, Sordaria*, and *Mortierella*. It is worth noting that some species of *Sphingomonas, Ohtaekwangia, Podospora*, and *Mortierella* play an important role in improving plant growth, enhancing resilience to plant pathogens and producing a variety of carbohydrate-active enzymes (Silar, 2013; Asaf et al., 2020; Ozimek and Hanaka, 2020; Deng et al., 2021). In our previous studies, four fungal and five bacterial strains were isolated from new reclamation soil, and those strains had a great ability to improve the soil fertility and promote plant growth by solubilizing P, fixing N, producing siderophores, and indole acetic acid (Li et al., 2021a,c). Those microbes may have greater potential to colonize and affect soil physicochemical properties and microbial communities in reclaimed barren soil.

To test whether some soil physicochemical properties influenced microbial composition dispersion, we used RDA to examine the correlation between microbial communities and environmental factors. The results indicated that a total of 53.42% of the cumulative variance of the root-zone bacterial community-factor correction was at the genus level, and the bacterial communities could be significantly influenced by available K, OMC, available P, and MBN (explained as 25.64%, 25.36%, 25.15%, and 23.38% of the bacterial community, respectively) in all four different treatments. Additionally, a total of 47.40% of the cumulative variance of the root-zone fungal community-factor correction at the genus level, and the fungal communities could be significantly influenced by available K, pH, and MBC (explained as 32.96%, 23.49%, and 22.67% of the fungal community, respectively) in all four different treatments. Previous studies have also revealed that the growth of soil microbes could be affected by a variety of environmental factors. For example, Li et al. (2023) found that the growth of pakchoi soil microbes was affected by available P, AHN, pH, OMC, and total N; Tian et al. (2017) reported that soil organic content exerted the largest effect on the distribution of bacterial communities.

Correlation networks of co-occurring microorganisms allow the visual summary of lots of information, and co-occurrence networks offer new insights into microbial interaction analysis (Chaffron et al., 2010; Jiang et al., 2022). In this study, we applied correlation-based network results to analyze the interaction between OTUs of different treatments (Sparcc's correlation *N* > 0.5 or <−0.5, *p* < 0.01). It is well-known that more nodes and edges and higher community diversity represent a complex and stable network structure (Hernandez et al., 2021), and high modularity is also an indicator of the network's structural stability (Ma et al., 2021). In our study, four different treatments had different microbial co-occurrence network structures. However, compared with the control, in the SMOF treatment, the number of bacterial network nodes increased, but the edges, the average degree, and the modularity decreased; meanwhile, the number of fungal network nodes, edges, and the average degree decreased, but the modularity increased. Strikingly, most of the relationships between the bacterial and fungal communities in the SMOF treatment were positive, which indicated that most bacteria and fungi had similar guilds or niches and were mutually beneficial rather than competitive (Deng et al., 2016).

A total of 1,054 peaks were screened from maize root-zone soil in four different treatments. The results of OPLS-DA revealed that the metabolites in control root-zone soil were significantly changed by SMOF, COF, and CCF, which was also verified by the volcano plot. A total of 277 metabolites were identified by LC-MS analysis, which mainly belongs to lipids and lipid-like molecules, organoheterocyclic compounds, benzenoids, organic acids and derivatives, organic oxygen compounds, and so on. In the SMOF and the control groups, there were 1,221 DEMs screened, with 1,107 upregulated and 114 downregulated. Compared to the control, 34 DEMs were significantly changed by SMOF, among which 10 DEMs (S-Nitrosoglutathione, edulan I, tetramethylpyrazine, 8,15-DiHETE, hypogeic acid, rotenone, 2-Pyrrolidinone, daucol, N-[(4-Hydroxy-3-methoxyphenyl)methyl]octanamide, [2,2-Bis(2-methylpropoxy)ethyl]benzene) were significantly upregulated, and five DEMs (stearidonic acid, (10E,12Z)-(9S)-9-Hydroperoxyoctadeca-10,12-dienoic acid, 9-HOTE, (3beta,5beta,8beta,22E,24xi)-Ergosta-6,22-diene-3,5,8-triol, Na,Na-Dimethylhistamine) were significantly downregulated. As we know, benzenoids, lipids, organoheterocyclic compounds, organic acids, phenylpropanoids, polyketides, and other secondary metabolites are essential to life, playing a vital role in the metabolism of all living cells. Previous reports have shown that a number of different benzenoid compounds are rapidly produced in plants or microbes in response to insects, pathogens, or stress (Keen and Taylor, 1975; Herrmann, 1995); lipids are essential for the integrity of cells and organelles by acting as a hydrophobic barrier for the membrane (Kim, 2020); organic acids play significant and varied roles in rhizosphere acidification and mineral weathering, contributing protons and serving as ligands for complex metals; they can promote redox reactions with electron-deficient metals (a rhizosphere-promoted process considered in the next section on redox cycling) (Daniel et al., 2007). To further explore the correlation between microbes and DEMs, a clustering heat map was drawn. The results showed that in the groups of SMOF and the control, there were four DEMs significantly correlated with two genera of bacteria, among which *Saccharibacteria* was significantly negatively correlated with two DEMs and positively correlated with one DEM, and *unclassified_Rhizobiales* was significantly negatively correlated with one DEM; meanwhile, 10 DEMs significantly correlated with five genera of fungi, among which *Cheilymenia* was significantly positively correlated with one DEM, *Paraphoma* was significantly negatively correlated with two DEMs, and positively correlated with one DEM, *Sodiomyces* was significantly positively correlated with one DEM, *Dipodascus* was significantly positively correlated with one DEM, and negatively correlated with four DEMs, and *Cephalotrichum* was significantly negatively correlated with four DEMs. The results revealed complicated interactions between microbes and DEMs in maize root zone soil.

## 5. Conclusions

In conclusion, our results showed that SMOF not only significantly improved the soil quality (including the soil's physical, chemical, and biological properties) but also promoted maize growth. This suggests that SMOF can be a good amendment for maize production in a reclaimed barren mountainous land. Compared with the control, SMOF caused a differential change in the microbial community of reclaimed barren mountainous land. Some specific beneficial microbes, such as *Ohtaekwangia, Sphingomonas, Sphingomonadacea, Saccharibacteria, Podospora*, and *Mortierella*, have been found to be closely related to the soil improvement by SMOF, indicating the relationships among microbes, fertilizer, and soil. Furthermore, RDA results showed that the composition of bacterial and fungal communities in maize root-zone soil was significantly affected by available K, OMC, P, MBN, and available K, pH, and MBC, respectively. In SMOF treatment, most microbes had similar guilds or niches and were mutually beneficial rather than competitive. In addition, SMOF resulted in a significant change in the kinds and relative contents of metabolites in the root-zone soil. The correlation heat map showed a significant correlation at the genus level between related microbial groups and DEMs of maize root-zone soil in SMOF. All these results revealed that SMOF could influence the interaction among soil properties, microbial communities, and secondary metabolites and improve maize growth. We believe that these may play an important role in improving soil quality and promoting maize production in the reclaimed barren mountainous land.

## Data availability statement

The datasets presented in this study can be found in online repositories. The names of the repositories are NCBI and CNGBdb, with accession number PRJNA951893, PRJNA951350 and CNP0004202, respectively.

## Author contributions

Conceptualization: XL, DW, QL, ZT, and JY. Methodology and investigation: XL, DW, and JY. Software: XL. Validation: XL, QL, ZT, and JY. Formal analysis, resources, and visualization: XL, QL, and JY. Data curation, supervision, project administration, and funding acquisition: QL and JY. Writing—original draft preparation and writing—review and editing: XL and JY. All authors have read and agreed to the published version of the manuscript.
